# Providing 2 Types of mHealth Interventions to Support Self-Management Among People Living With HIV: Randomized Clinical Trial

**DOI:** 10.2196/60905

**Published:** 2025-05-29

**Authors:** Gwang Suk Kim, Layoung Kim, Seoyoung Baek, Sooyoung Kwon, Ji Min Kim, Jun Yong Choi, Jae-Phil Choi

**Affiliations:** 1 Mo-Im Kim Nursing Research Institute, College of Nursing, Yonsei University, 50-1, Yonsei-Ro, Seodaemun-gu, Seoul, 03722, Republic of Korea, 82 1088182364; 2 College of Nursing and Brain Korea 21 FOUR Project, Yonsei University, Seoul, Republic of Korea; 3 Korea Armed Forces Nursing Academy, Daejeon, Republic of Korea; 4 Department of Nursing, Graduate School of Yonsei University, Seoul, Republic of Korea; 5 Division of Infectious Disease, Yonsei University Health System, Seoul, Republic of Korea; 6 Department of Internal Medicine, College of Medicine, Yonsei University, Seoul, Republic of Korea; 7 Division of Infectious Diseases, Seoul Medical Center, Seoul, Republic of Korea

**Keywords:** telemedicine, HIV, self-management, mHealth, randomized clinical trial, mobile health

## Abstract

**Background:**

Mobile health (mHealth) has been continuously developed to support the HIV care continuum for people living with HIV. Considering the practical needs and acceptability of digital health solutions, it is essential to explore effective content and diverse delivery methods for self-management support.

**Objective:**

This study aimed to assess the effectiveness of 2 non–face-to-face mHealth interventions for people living with HIV. We compared the impact on HIV self-management of (1) a link group, which received access to information via mobile link, and (2) an app group, which used a mobile app enabling information exploration and self-recording of health outcomes, including medication adherence, symptoms, mental health score, and sexual safety.

**Methods:**

A 2-arm, prospective, randomized clinical trial was conducted, involving 83 people living with HIV aged 19 years or older, who were assigned to the app group (n=42) or link group (n=41). The primary outcome was self-reported self-efficacy for HIV management (HIV-SE), which comprised 6 domains: managing depression or mood, medication, symptoms, and fatigue; communicating with health care providers; and getting support or help. A paired *t* test and generalized estimating equation were used to analyze the outcomes at baseline, 4 weeks postintervention, and 8 weeks after an additional 4-week voluntary use period.

**Results:**

Both groups demonstrated improvements in total HIV-SE scores at 4 weeks compared with baseline. All domain scores improved in the app group, with a significant increase in total HIV-SE and managing fatigue. The link group significantly improved in managing depression or mood, fatigue, and getting support or help domains. The generalized estimating equation analysis indicated that, compared with the link group, the app group had significant group-by-time interaction with a positive effect on managing symptoms at 4 weeks (β=0.635, 95% CI 0.023 to 1.247; *P*=.04) but a negative effect on managing depression or mood at 8 weeks (β=−0.824, 95% CI −1.448 to −0.200; *P*=.01). Only 9.5% (4/42) of app group participants maintained daily visits during the voluntary use period of 4 to 8 weeks.

**Conclusions:**

Both types of informational mHealth interventions, through mobile apps or link access, contributed to improving HIV-SE. Delivering information via direct text message links could be suitable for individuals who are hesitant to use HIV-related apps. While mobile apps promote self-monitoring and symptom management through self-recording and reflection, strategies are needed to sustain long-term app engagement. In addition, user-customized psychiatric content beyond mental health recordings has been suggested for managing depressed moods in mHealth interventions.

## Introduction

People living with HIV who adhere to antiretroviral therapy (ART) and achieve viral suppression have not been reported to sexually transmit the virus to others [1]. According to a cohort study by the Korea Disease Control and Prevention Agency, the ART adherence rate in Korea, defined as taking medication without interruption for 2 consecutive weeks, was 87.6% [2,3]. Additionally, national HIV epidemiology data reported that the ART treatment rate, based on prescription records in Korea, was 95.5%, and the viral suppression rate was 96%, indicating that most people living with HIV belong to the retention in care [4]. While scientific knowledge of “undetectable equals untransmittable (U=U)” has contributed to transforming HIV into a chronic illness, HIV-related discrimination with stigma remains prevalent in health care settings [5]. People living with HIV rely on infectious disease hospital-based resources for HIV-related information and consultation [6]. Several mobile-based interventions have been developed to address barriers to taking medications or visiting clinics, offering features such as adherence tracking and text message reminders [7,8]. However, existing mobile health (mHealth) solutions primarily focus on improving ART adherence rather than addressing the needs of the majority of people living with HIV who are already adherent to ART and retained in care. Moreover, studies have reported that the adoption and use of apps account for only a small proportion [9,10]. Despite the increasing availability of digital health information, this population continues to express a need for evidence-based, reliable information in the mHealth needs survey [11]. These findings highlight the ongoing demand for informational mHealth solutions for community-dwelling people living with HIV.

Given that many people living with HIV achieve ART adherence, integrated care to empower them to play a role in the HIV care continuum is increasingly emphasized [12,13]. Similar to other chronic illnesses, they require self-efficacy to manage both their HIV condition and holistic health beyond HIV disease in a long-term manner [13]. Self-efficacy for HIV disease management is a perceived behavior control indicator that practices health care behaviors along with compliance with treatment. Therefore, it is often used to evaluate the outcome of integrated chronic disease management, beyond clinical parameters such as viral suppression [14]. Integrated health services should encompass not only ART management but also engaging in social life, family planning, maintaining a safe sexual life, and managing psychological health, aligning with the vision of the HIV care continuum. In this study, informational mHealth interventions comprised 6 domains—HIV management, ART care, psychological health care, sexual health care, health promotion, and family and social life—that could provide knowledge and information regarding integrated health services [11,12]. Informational mHealth was designed to be delivered through either a mobile app or direct links via text messages. While mobile apps offer a structured platform to improve self-efficacy in HIV management, concerns over privacy and fear of disclosing HIV status may pose barriers to adoption. The coexistence of concealment and exposure [15] should be ensured and understood in the context of mHealth interventions. Considering differences in user preferences, whether receiving information solely through text message links is effective needs to be evaluated. Furthermore, the potential advantages of mobile apps—not only as information sources but also for their self-recording functionalities—should be explored. This study evaluates the effectiveness of informational mHealth in improving self-efficacy for HIV management (HIV-SE). We assess the impact of 2 mHealth delivery methods—a mobile app and links in text messages—through a prepost study design and compare their effectiveness in improving self-efficacy among people living with HIV.

## Methods

### Ethical Considerations

This study was approved by the Institutional Review Board of the first author’s affiliation (4-2022-1192), and the protocol was registered with the Clinical Research Information Service (KCT0008014). All interventions and assessments were conducted through non–face-to-face contact, maintaining the privacy and confidentiality of the participants. Self-reported assessments were administered only after participants read and clicked on the informed consent via a web-based platform. Participants self-downloaded the mobile app from Google Play or the App Store and registered it on their mobile device. The app was available for both iOS and Android platforms, ensuring accessibility and minimizing potential selection bias in participation. Participants took part anonymously and could withdraw from the study at any time by simply choosing not to respond. To protect confidentiality, the app used a secure user authentication system that did not require personally identifiable information, using an anonymized ID and password. All self-recorded data, including diary entries and symptom tracking, were stored in a deidentified format. Users also had the option to delete their records at any time, giving them full control over their data. After completing the interventions, participants were compensated with gift coupons worth US$ 70 (KRW ₩100,000) for the app group and US$ 35 (KRW ₩50,000) for the link group.

### Trial Design

**Figure 1.**
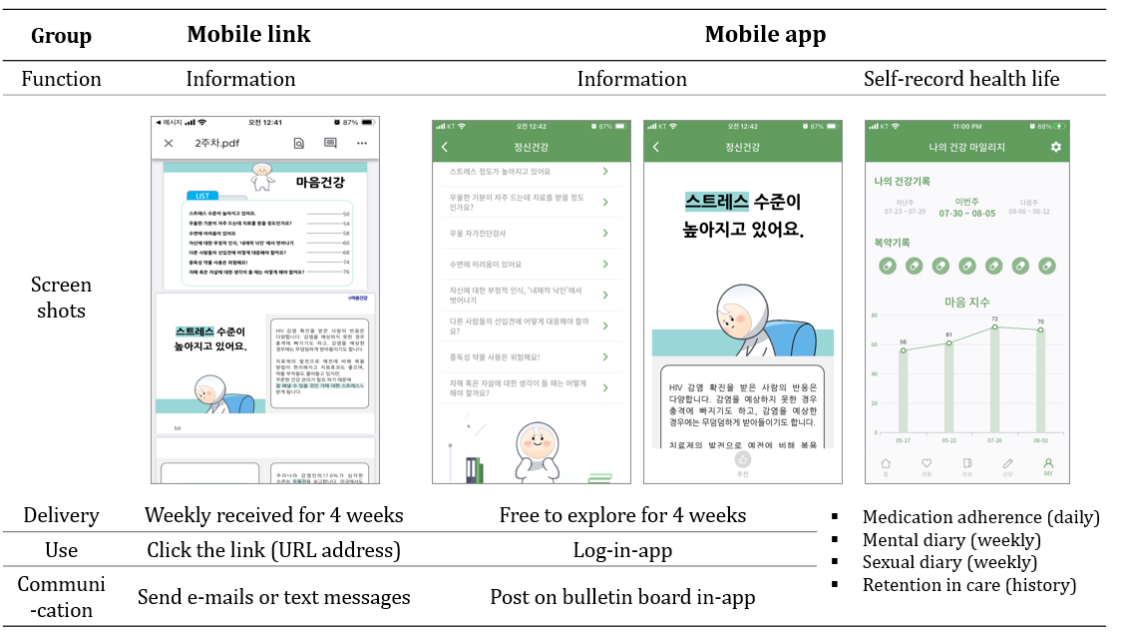
Overview of mobile intervention methods: mobile link versus mobile app.

This study was a 2-arm randomized clinical trial. We compared the link group, which received text messages of URL links for accessing information, with the app group, which used a mobile app for exploring information and self-recording health outcomes (Figure 1). The 2 interventional arms were implemented using participants’ smartphones. The contents of the information provided were the same in both groups; however, the delivery methods were different. The link group clicked on the URL address of the weekly received text messages, and the app group logged into the app to view the content posted at all times.

A priori sample size was estimated using G*power 3.1.9.2 software. Based on a previous study evaluating a similar mHealth intervention [16], a sample size of 60 participants was required to detect an effect size of 0.44, with a type 1 error rate of 5% and a power of 0.80. Considering an expected dropout rate of approximately 30%, the target sample size was set at 86 participants. Block randomization (block size of 4) was used to ensure 1:1 balanced enrollment. Research staff who generated the random numbers and block sequences (ie, AABB, ABAB, BAAB, BABA, BBAA, and ABBA) using Microsoft Excel and those who registered participants were different. However, the research staff could not be blinded after the arm assignment to be involved in data control. Participants were unable to blind the assignment in the app-based trial. The research staff and participants did not know each other before and had never met. All contacts from the research team were made via text messages or email, and a phone number was provided for participants to call 24/7 if needed. The period of the mHealth intervention was 4 weeks, with assessments at baseline (T0), 4 weeks postintervention (T1), and a follow-up at 8 weeks, after an additional 4-week voluntary use period (T2).

### Participant Recruitment

The participants were recruited between July 19 and October 18, 2023. The eligibility criteria were as follows: (1) people diagnosed with HIV, aged 19 years and older, (2) individuals who owned and used a smartphone, and (3) those who had no difficulty reading and responding to the questionnaire via smartphone. Participants were excluded if they had a medical diagnosis that required immediate treatment and counseling, other than HIV infection. Participants were recruited using an online access method. A prior study to identify the mHealth needs of people living with HIV was conducted at 5 hospitals, and about half of the participants expressed their willingness to participate in further mHealth interventions. A recruitment poster was emailed to 138 individuals who had left their email addresses and expressed interest in participating in the intervention study based on a previous service-needs assessment conducted in 2020‐2021. In addition, 340 small leaflets that fit into a pocket were distributed to visiting outpatients “people living with HIV” by medical staff at the 2 hospitals. The poster and leaflets specified the study purpose and participation method, and included a QR code that could access the web-based baseline survey. Participants who completed the baseline survey were randomly assigned to the app or link groups. The research staff sent the app group a text message explaining how to download the app from the App Store or Google Play. The app allowed participants to log in only after receiving approval from the research team. The link group received a text message mentioning that they would receive a weekly text message including a link to access the information.

### Interventions

#### Overview

Both groups received text messages from the research team once a week to encourage their engagement with mHealth during the 4 weeks of intervention. The app group received a text message that encouraged the use of app functions, including the information screen, while the link group was encouraged to read the information material. After completing the intervention period at 4 weeks (T1), the participants were informed that the mandatory use period ended and that they could use the app or link address autonomously for the next 4 weeks (T2). During the 4 weeks of voluntary use, the research team did not contact the participants.

#### App Group

##### Overview

This group used a mobile app to support their HIV self-management. The app, namely, “ESSC (Excellent Self-Supervised HIV Care),” was developed using 3 iterative cycles of the Design Science Research framework [11]. Three cycles comprise relevance, derived from users’ requirement need survey; rigor, organized based on the information–motivation–behavioral skills model with validity being evaluated by experts; and design, which builds artifacts including functions, contents, and features. Formative details of the development process are described in a previous study [11]. The major functions of the ESSC app are as follows.

##### Information

Overall, 47 health information components were provided in 6 domains: HIV management, ART care, psychological health care, sexual health care, health promotion, and family and social life. Integrated information was created to provide essential information about leading a healthy life with HIV. Each piece of information includes a literature source for accuracy and trustworthiness. To deliver acceptable and intensive information, all content was designed with brief descriptions and images, such as poster presentation templates. The title of each was framed as a question (eg, Would it be a problem if I do not notify others that I am infected?).

##### Health Life Records

Users record their health in 4 categories: medication adherence, mental diary, sexual diary, and retention in care. They checked the boxes of daily ART medication adherence and uncomfortable symptoms. In the mental diary, users could choose their strengths weekly, which were presented as 24 items by Peterson and Seligman [17]. After being aware of the strength, negative and positive emotions experienced last week were recorded and scored as an overall emotional score (−100=as sad as I can imagine, 0=not sad at all, 100=as happy as I can imagine). In the sexual diary, users could record sexual activity within the past week and check boxes for any difficulties they experienced in their sexual life (disclosing HIV to a partner, using condoms, fear of viral detection, believing that people living with HIV cannot have sex, etc). Then, they identified safe sexual guidelines for people living with HIV. For retention in care, the user was asked to record clinic visits and the progress of laboratory results (CD4+T lymphocyte [CD4+T] cell count and viral load).

##### Others

Users’ health records are demonstrated by graphical and descriptive presentations on “My page.” Self-reflection on one’s own health records enables individuals to be aware of meaningful changes and motivates them to engage in healthy behaviors [18]. After completing the mental and sexual diaries, a pop-up message prompted users to communicate with the health care provider (HCP). When users left various questions about information, personal concerns, and app features, a nurse with more than 10 years of experience in HIV research responded within 24 hours.

### Link Group

These participants received text messages, including the link to access information, once a week for the 4-week intervention period. When participants click the URL address in the text message, the screen is linked to the educational material, a PDF file. This information is the same as in the poster-templated content of the ESSC app. The 6 domains were categorized into 4 sessions: HIV management, psychological and sexual health care, ART care, and health promotion and family and social life. When participants had questions about information or personal concerns, they could send text messages or emails to the research team.

### Measurement

#### Overview

Participants’ basic information was measured at baseline (T0), and primary and secondary outcomes were assessed 3 times (T0, T1, and T2). The usability of the ESSC app was evaluated at 4 weeks (T1). All assessments were self-reported using a web-based platform (Google Survey).

#### Primary Outcome: Self-Efficacy for HIV Management

The Korean version of the HIV-SE scale [14] was used. The 33 items comprised 6 domains: managing depression or mood (9), medications (6), symptoms (5), and fatigue (4); communicating with the HCP (4); and getting support or help (5). The participants rated confidence in their ability to perform specific behaviors on a 10-point Likert scale (1 = “not sure at all” to 10 = “totally sure”). The original scales developed by Shively et al [19] were translated into Korean and validated the conceptual domains as a 6-factor model. In previous studies [20-23], the 6 domains were used to identify specific subconcepts of self-efficacy and analyze the multivariate factors in predicting self-management. Cronbach α for the Korean scale was 0.91 [14] and 0.93 in this study.

#### Secondary Outcomes

Medication adherence was measured using a self-reported visual analogue scale (VAS), where participants were asked to rate their adherence to prescribed medication over the past month on a scale from 0 to 100 (0=not taking the prescribed medications, 100=taking every single dose of prescribed medications). This method has been used in previous adherence studies [24,25] and has shown a strong correlation with objective adherence measures such as electronic monitoring [25]. A single-item VAS was selected to avoid redundancy, as adherence behaviors were already assessed through the HIV-SE domain “managing medications,” which includes taking medications on time, daily, and according to prescribed instructions.

Self-efficacy regarding condom use was assessed using a single item on perceived behavioral control toward safe sexual intercourse. “If I have sex with my partner, I am confident that I always use a condom,” the participants responded on a 5-point Likert scale (1 = “not at all” to 5 = “very much”).

Health-related quality of life was assessed using the EuroQoL group’s VAS, which measures perceived health on a particular day on a scale ranging from 0 to 100 (0=the worst health imaginable, 100=the best health imaginable) [23].

#### Usability Tests

Usability tests were conducted only for the app group. User records accumulated in the app were used to quantitatively assess usability. The Health Information Technology Usability Evaluation Scale (Health-ITUES) was used to evaluate the quality of the ESSC application. Schnall et al [26] developed 20 items of Health-ITUES to evaluate the usability of a mobile app for symptom self-management of community-dwelling people living with HIV on a 5-point Likert scale (from 1 = “strongly disagree” to 5 = “strongly agree”). The Korean version [27] was adapted for HIV disease self-management and includes 4 subscales: impact, perceived usefulness, ease of use, and user control. Finally, the participants were free to write about any problems they experienced while using the app or anything that needed to be corrected or supplemented. This qualitative feedback was also conducted in the link group.

#### Basic Information

Sociodemographic variables included age, sex, educational level, economic status, marital status, residence, employment status, and gender orientation. Use and need for mHealth were assessed by responses to the time spent using mHealth per day and their requirements for HIV health apps on a 10-point Likert scale (1 = “not at all” to 10 = “totally need”). The Short-Form Health Literacy Scale (SF-HLS) was used to measure participants’ literacy, including access to and understanding of health care, disease management, and health promotion. The Korean version [28] that verified the reliability and validity of the original 12 items [29] was used.

For personal HIV status, data on the year of diagnosis, HIV status with symptoms, transmission mode of infection, and experience with self-help participation were collected. The Korean version [30] of the Short Form of the World Health Organization Quality of Life–HIV questionnaire (WHOQoL-HIV-BREF) [31] was partially used to assess the psychological domain. Laboratory results for viral load and CD4+T cells were self-reported based on participants’ most recent hospital visit. Viral load was classified as undetectable, detectable, or unknown, and CD4+T cell counts were classified into ≥500, 200‐499, <200 cells/mm^3^, and unknown [32]. In addition, participants were asked whether they had taken medication without interruption for 2 consecutive weeks, using the same approach as the Korea Disease Control and Prevention Agency cohort study [3], to allow for comparison of baseline adherence levels with the general population of people living with HIV.

### Statistical Analysis

Basic information variables at baseline are presented by the groups using descriptive analysis (counts with percentages or mean values with SDs). Changes in primary and secondary outcome variables between baseline and postintervention were analyzed using paired 2-sample *t* tests, which examined the differences in the mean values of outcomes by group during the intervention. The primary outcome variables were compared between the app and link groups using a generalized estimating equation (GEE). It is an appropriate statistical method that analyzes repeated measures data reflecting the interaction between group and time and performs intention-to-treat analysis. To compare the effectiveness of group differences across time, GEE analysis was performed, including the results of the 3 assessment points (T0, T1, and T2). Here, the GEE analysis was adjusted for 3 covariates: number of years after HIV diagnosis, psychological domain of the WHOQoL-HIV BREF, and SF-HLS. Previous studies have shown that the HIV self-management ability was affected by the duration of HIV diagnosis, psychological status, and literacy [33-36]. In addition, health literacy scores were significantly different between the 2 groups in the homogeneity test of basic information using the chi-square test or independent *t* test. Therefore, the research team acknowledged that these 3 covariates should be included in the evaluation of HIV-SE effectiveness. For all analyses, the threshold for statistical significance was a *P* value of .05. According to the statistical method, the GEE analysis included all data of the withdrawn participants, but the paired *t* test could not include withdrawn participants. Additionally, open-ended qualitative feedback collected during the usability test was analyzed using a descriptive content analysis approach. Two researchers independently reviewed the responses, grouped them into common themes, and categorized the feedback as either positive or negative from the user perspective. Key patterns were summarized narratively to complement the quantitative usability findings.

## Results

### Participants

**Figure 2.**
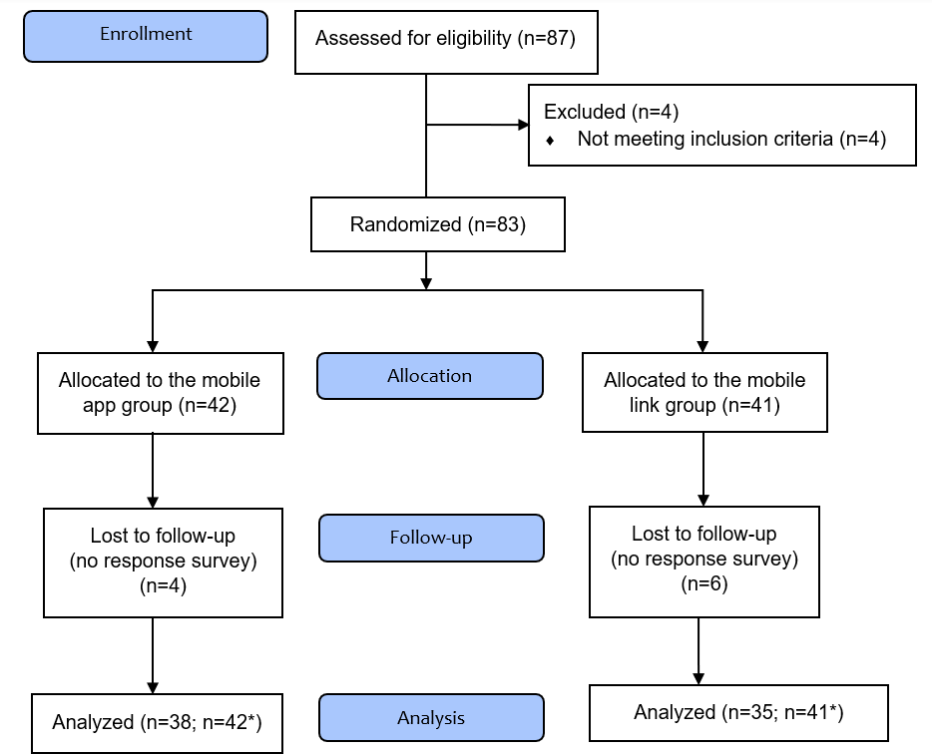
Consolidated Standards of Reporting Trials (CONSORT) flow diagram for trial recruitment. *Data were analyzed using a generalized estimating equation.

Figure 2 illustrates a flow diagram for trial recruitment. Overall, 87 individuals accessed online sites to participate. After responding to the baseline survey, 4 were excluded as they met the exclusion criteria: had a psychiatric diagnosis (ie, bipolar disorder or depression) with symptoms, and were under treatment. The remaining 83 people living with HIV were assigned to the 2 groups, of which 73 completed the 8-week follow-up. When required to self-report assessments after completing the intervention at 4 weeks, 12% (10/83) of participants did not respond to the research team contact.

The baseline characteristics are shown in Table 1. The mean age of the participants was 39.42 years (range: 21‐69 years), 96.4% (80/83) men, and the majority, 85.5% (71/83), were men who had sex with men, including homosexual and bisexual gender orientation. A total of 9 in 10 participants had never been married, and half lived alone. There was an average of 9.51 years after HIV diagnosis, and 6 in 7 participants had viral suppression and no HIV-related symptoms. Only 7 perceived themselves as having an AIDS status. The results of the homogeneity test showed that the SF-HLS scores (*t*(81)=2.667, *P*=.009) were significantly different between the 2 groups, while the other variables showed no significant differences.

**Table 1.** Baseline characteristics of the study participants.

Variable	Total (n=83)	App group (n=42)	Link group (n=41)
Age (years), mean (SD)	39.42 (10.38)	37.67 (10.72)	41.22 (9.82)
Sex, n (%)			
Male	80 (96.4)	40 (95.2)	40 (97.6)
Female	3 (3.6)	3 (4.8)	1 (2.4)
Education level, n (%)			
≤High school	32 (38.6)	20 (47.6)	12 (29.3)
≥College	51 (61.4)	22 (52.4)	29 (70.7)
Economic status, n (%)			
High	5 (6.0)	3 (7.1)	2 (4.9)
Middle	47 (56.6)	21 (50.0)	26 (63.4)
Low	31 (37.4)	18 (42.9)	13 (31.7)
Marital status, n (%)			
Never married	74 (89.2)	39 (92.9)	35 (85.3)
Married	7 (8.4)	3 (7.1)	4 (9.8)
Divorce	2 (2.4)	0 (0.0)	2 (4.9)
Residence, n (%)			
Alone	45 (54.2)	26 (61.9)	19 (46.3)
With family	26 (31.3)	13 (31.0)	13 (31.7)
With friend	12 (14.5)	3 (7.1)	9 (22.0)
Employment, n (%)			
Not working	17 (20.5)	10 (23.8)	7 (17.1)
Employed (temporary)	18 (21.7)	9 (21.4)	9 (21.9)
Employed (regular)	48 (57.8)	23 (54.8)	25 (61.0)
Gender orientation, n (%)			
Homosexual	62 (74.7)	30 (71.4)	32 (78.1)
Heterosexual	12 (14.5)	6 (14.3)	6 (14.6)
Bisexual	9 (10.8)	6 (14.3)	3 (7.3)
Mobile health, mean (SD)			
Need (out of 10 points)	7.04 (2.43)	7.17 (2.58)	6.90 (2.30)
Use (hour/day)	4.28 (3.41)	4.30 (4.09)	4.27 (2.58)
Short-form health literacy, mean (SD)			
Scale (out of 4 points)	3.04 (0.62)	3.22 (0.55)	2.87 (0.65)
Years after diagnosis^a^	9.51 (7.04)	8.83 (7.45)	10.27 (7.68)
HIV status, n (%)			
No symptoms	72 (86.8)	37 (88.1)	35 (85.4)
Have symptoms	4 (4.8)	2 (4.8)	2 (4.9)
AIDS	7 (8.4)	3 (7.1)	4 (9.7)
Visit a hospital within 6 months, n (%)	79 (95.2)	40 (95.2)	39 (95.1)
2-week uninterrupted ART^b^, n (%)	65 (78.3)	36 (85.7)	29 (70.7)
CD4+T^c^ cell count, n (%)			
<200	5 (6.0)	2 (4.8)	3 (7.3)
200~499	13 (15.7)	9 (21.4)	4 (9.8)
500 or over	57 (68.7)	25 (59.5)	32 (78.0)
Unknown	8 (9.6)	6 (14.3)	2 (4.9)
Viral load, n (%)			
Undetectable	71 (85.6)	37 (88.1)	34 (82.9)
Detectable	6 (7.2)	1 (2.4)	5 (12.2)
Unknown	6 (7.2)	4 (9.5)	2 (4.9)
WHOQoL-HIV BREF^d^, mean (SD)			
Psychological domain	61.30 (14.04)	64.19 (14.00)	58.34 (13.62)
Transmission mode, n (%)			
Heterosexual intercourse	7 (8.4)	2 (4.7)	5 (12.2)
Homosexual	58 (69.9)	33 (78.6)	25 (61.0)
Injection of blood, etc	18 (21.7)	7 (16.7)	11 (26.8)
Self-help group participation, n (%)	19 (22.9)	12 (28.6)	7 (17.1)

a In the mobile link group, 4 data points were missing because the participants did not remember their diagnosis year.

b ART: antiretroviral therapy.

c CD4+ T: CD4 T lymphocyte.

d WHOQoL-HIV BREF: World Health Organization Quality of Life–HIV questionnaire.

### Outcomes

The difference in mean values of each group of primary and secondary outcomes between baseline (T0) and postintervention (T1) is shown in Table 2. During the intervention period, the overall outcomes improved in the app group, except for medication adherence. The results of the paired *t* test showed that the total score and the managing fatigue domain of the HIV-SE significantly improved in the app group. The link group also showed significant improvements in 3 domains: managing depression or mood, managing fatigue, and getting support or help. However, there was no significant improvement in secondary outcomes in either group. At 8 weeks (T2), the voluntary use for 4 weeks after completing the intervention, the overall primary outcomes decreased from the measured score at T1 (Table 3), but managing depression or mood in the link group increased over 8 weeks.

**Table 2.** Differences in the mean of outcomes during the intervention period.

Variables	App group (n=38), mean (SD)			Link group (n=35), mean (SD)		
	T0^a^	T1^a^	*P* value^b^	T0	T1	*P* value^b^
Self-efficacy for HIV self-management	6.82 (1.51)	7.19 (1.37)	.03	6.37 (1.27)	6.61 (1.31)	.07
Managing depression or mood	6.47 (1.97)	6.72 (1.78)	.36	5.57 (1.49)	6.03 (1.63)	.04
Managing medication	8.61 (1.80)	8.74 (1.45)	.65	8.67 (1.25)	8.81 (1.10)	.28
Managing symptoms	7.40 (2.28)	7.94 (1.52)	.08	7.24 (1.59)	7.21 (1.86)	.47
Managing fatigue	6.60 (2.31)	7.32 (1.92)	.03	5.74 (2.03)	6.21 (2.00)	.02
Communication with HCP^c^	7.92 (2.18)	8.03 (2.11)	.95	7.76 (2.35)	7.53 (2.14)	.35
Getting support or help	4.02 (2.13)	4.64 (2.55)	.10	3.56 (1.82)	3.97 (1.98)	.03
Medication adherence	95.50 (16.31)	94.71 (16.97)	.58	97.17 (5.26)	97.23 (5.77)	.56
Self-efficacy about condom use	3.64 (1.41)	3.97 (1.17)	.24	3.41 (1.18)	3.46 (1.22)	.40
Health-related quality of life	72.69 (15.31)	76.11 (18.89)	.16	73.37 (13.38)	73.77 (20.35)	.93

a T0=baseline; T1=4 weeks follow up.

b Results based on paired *t* test.

c HCP: health care provider.

**Table 3.** Comparison of self-efficacy for HIV self-management between the mobile app and link group.

Variable	App group (n=42), mean (SD)	Link group (n=41), mean (SD)	App group*Time interaction effects	
			β (95%CI)	*P* value^a^
Self-efficacy for HIV self-management				
T0^b^	6.82 (1.51)	6.37 (1.27)	—^c^	—
T1^b^	7.19 (1.37)	6.61 (1.31)	0.029 (−0.301 to 0.358)	.86
T2^b^	6.83 (1.42)	6.53 (1.38)	−0.264 (−0.649 to 0.120)	.18
Managing depression or mood				
T0	6.47 (1.97)	5.57 (1.49)	—	—
T1	6.72 (1.78)	6.03 (1.63)	−0.204 (−0.755 to 0.347)	.47
T2	6.38 (1.95)	6.30 (1.55)	−0.824 (−1.448 to −0.200)	.01
Managing medication				
T0	8.61 (1.80)	8.67 (1.25)	—	—
T1	8.74 (1.45)	8.81 (1.10)	−0.219 (−0.612 to 0.174)	.27
T2	8.75 (1.51)	8.49 (1.32)	0.051 (−0.433 to 0.536)	.84
Managing symptoms				
T0	7.40 (2.28)	7.24 (1.59)	—	—
T1	7.94 (1.52)	7.21 (1.86)	0.635 (0.023 to 1.247)	.04
T2	7.25 (2.26)	6.82 (2.09)	0.296 (−0.403 to 0.996)	.41
Managing fatigue				
T0	6.60 (2.31)	5.74 (2.03)	—	—
T1	7.32 (1.92)	6.21 (2.00)	0.073 (−0.531 to 0.678)	.81
T2	6.55 (2.52)	5.95 (2.06)	−0.346 (−1.110 to 0.417)	.37
Communication with HCP^d^				
T0	7.92 (2.18)	7.76 (2.35)	—	—
T1	8.03 (2.11)	7.53 (2.14)	0.099 (0.570 to 0.768)	.77
T2	8.13 (1.82)	7.70 (2.21)	−0.060 (−0.752 to 0.632)	.87
Getting support or help				
T0	4.02 (2.13)	3.56 (1.82)	—	—
T1	4.64 (2.55)	3.97 (1.98)	0.047 (−0.719 to 0.814)	.90
T2	4.24 (2.34)	3.83 (1.95)	−0.166 (−0.790 to 0.458)	.60

a Results based on generalized estimating equations adjusted for the duration of HIV infection, short-form health literacy scale, and psychological domain for the World Health Organization Quality of Life–HIV questionnaire (group: mobile link group reference; time points: T1 and T2, with T0 as reference).

b T0=baseline; T1=4 weeks follow up; T2=8 weeks follow up.

c Not applicable.

d HCP: health care provider.

GEE was used for group comparisons of differential changes from T0 at T1 and T2. The effects of the app group compared with the changes in the link group over time points, as a reference at T0 in self-efficacy for HIV self-management, are shown in Table 3. Regarding symptom management, the app group showed a significant improvement at T1, but this effect was not observed at T2. On the other hand, a significant reduction was observed at T2 in scores for managing depression or mood compared with the link group. There were no statistically significant findings for the other outcome variables in the GEE analysis.

### Usability Test of the Mobile App

**Figure 3.**
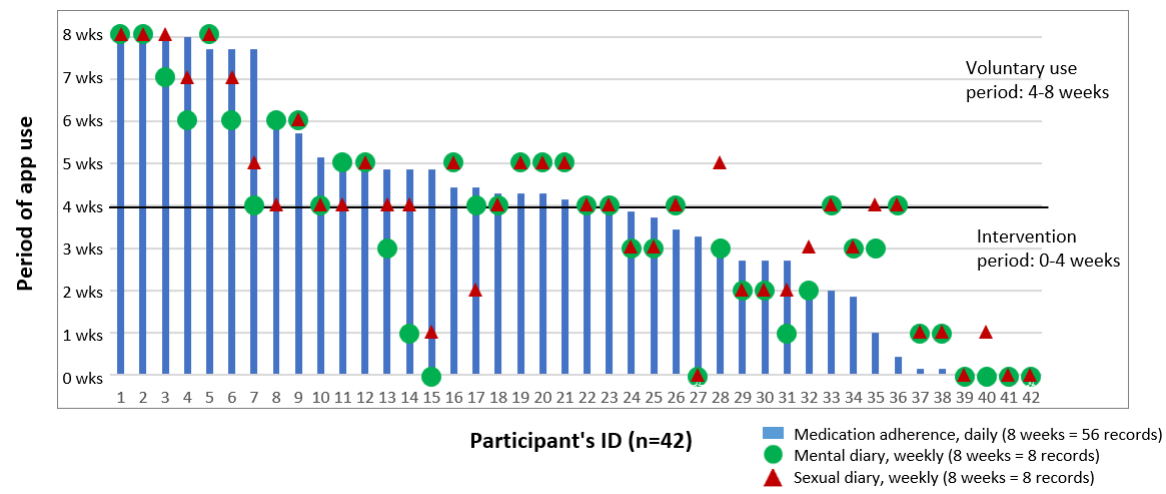
Graph of the app usage by mobile app participant ID over 8 weeks.

Regarding the quantitative usability of the ESSC app during the 4-week intervention period, 42.9% (18/42) of app group participants completed the in-app logs fully, with medication adherence at 54.8% (23/42), mental diary at 54.8% (23/42), sexual diary at 64.3% (27/42), and treatment retention at 97.6% (41/42) (Table 4). Only 9.5% (4/42) maintained daily medication records from the completion of the intervention to the voluntary use period of 4‐8 weeks. The periods of app use per participant ID are presented in Figure 3. Individuals who recorded daily medication adherence tended to record weekly mental and sexual diaries.

**Table 4.** Number of users and records of mobile app group during the intervention period (n=42).

Categories	Users, n (%)	Completers, n (%)	Records, n (%)
Medication adherence (daily)	40 (95.2)	23 (54.8)	877 (100)
Took ART^a^	40 (95.2)		855 (97.5)
Have symptoms	8 (19.0)		40 (4.6)
Sexual life (weekly)	38 (90.5)	27 (64.3)	128 (100)
Have sexual intercourse	19 (45.2)		31 (24.2)
If yes, condom usage	10 (23.8)		14 (10.9)
Mental diary (weekly)	36 (85.7)	23 (54.8)	120 (100)
Feeling sad	7 (16.7)		12 (10.0)
Retention in care (recent history)	41 (97.6)	41 (97.6)	45 (100)
Visit a hospital within 6 months	39 (92.9)		44 (97.8)
CD4+T^b^ cell (500 or over)	27 (64.3)		30 (66.7)
Viral load (undetectable)	33 (78.6)		36 (80.0)

a ART: antiretroviral therapy.

b CD4+T: CD4+T lymphocyte.

The mean scale of Health-ITUES after the intervention period was 3.98 (SD 0.75) out of 5 points. The 4 subscale scores were as follows: impact (3.93, SD 0.95), perceived usefulness (3.86, SD 0.99), perceived ease of use (4.32, SD 0.71), and user control (3.95, SD 0.80). While it was easy for the participants to become skillful users of the ESSC app, the impact on a healthy life and the usefulness of supporting HIV self-management did not exceed 4 points. Qualitative feedback regarding the ESSC app or the link is shown in Textbox 1. Participants believed that the information was easy to understand and important; they wanted to regularly receive updated information, such as up-to-date treatment (eg, long-acting injectable HIV ART).

Textbox 1. **Qualitative feedback after using the Excellent Self-Supervised HIV Care app or the link.**

**The Excellent Self-Supervised HIV Care app in the app group:**

A. Information

- Positive: “Easy to understand without difficult terms” and “Essential and comprehensive content”
- Negative: “Lack of up-to-date information (eg, latest treatment development trend, case of cure, and clinical study results),” “Lack of details and diversity (eg, specific opportunistic infections such as condyloma),” and “Need to upload new content each week”

B. Self-record health life

- Positive: “Care more about taking medicine than before”
- Negative: “Unnecessary for smart patients with good medication adherence” and “Mind diary only suitable for depressed people”

C. Engagement

- Positive: “A cute character” and “Appropriate for the initial stage”
- Negative: “Insufficient motivation to use” and “Need for community features to communicate between users”

**Accessing information in the link group:**

A. Information

- Positive: “Well-organized information” and “Accurate, reliable information”
- Negative: "Needs to be constantly updated," "Pursue more details," and "Enlarge health information beyond HIV treatment"

B. Digital resource

- Positive: “An opportunity to read and study on my own, unlike consulting with medical staff” and “Easy to remember like an ebook”
- Negative: “Too large volume to view on a mobile phone”

## Discussion

### Principal Results

This study is the first trial to conduct fully non–face-to-face digital interventions for community-dwelling people living with HIV in Korea. These digital interventions have been designed to expand access to health information resources that are limited to hospital settings. Two-arm interventions were performed using a mobile app or link in text messages, which are popular implementation methods for mHealth. Comprehensive information that helps people living with HIV self-manage their HIV disease and a healthy life was provided by logging into the app or accessing links in the text messages. While the mobile link provided only information, the mobile app had functions to self-record and view their recorded health data. After 4 weeks, the total self-efficacy score for HIV management increased from the baseline in both groups, and a statistically significant improvement was found in the app group. Although the GEE analysis revealed no significant between-group difference in total self-efficacy score over 8 weeks, domain-specific comparisons indicated that the app group experienced a significant positive effect in managing symptoms at 4 weeks and a negative effect in managing depression or mood at 8 weeks. Our findings suggest that the information-driven mobile app helps improve self-efficacy for HIV disease management and that the function of self-recording health life has benefits for symptom management.

### Informational mHealth Intervention Implementation

Informational support is the most frequently requested and provided form of assistance within online support groups [37]. In this study, 9 in 10 participants were single men, and half lived alone; they spent more than 4 hours a day using the app or accessing the internet via smartphone. Despite being well-positioned to engage in online support groups, only a quarter of participants had experienced participating in self-help groups. Previous studies on online-based relationships among people living with HIV have identified challenges such as difficulty maintaining relationships, concerns about HIV disclosure, and the spread of inaccurate information [38]. In line with a prior mHealth needs survey, it was found that people living with HIV desired evidence-based and trusted information first, and easy-to-understand content [11]. They reported that the need to communicate with other people living with HIV was low, but the need for HCP was high [11]. In this study, mHealth interventions through a mobile app or link provided validated information from HCP across 6 domains: HIV management, ART care, psychological health care, sexual health care, health promotion, and family and social life, which covered a holistic approach to healthy life. While both groups demonstrated improvements in total self-efficacy scores, no improvements were observed in the domains of managing medication or communication with HCP. Similarly, medication adherence did not improve, which aligns with previous studies showing that digital interventions often have limited impact on adherence among people living with HIV who are already taking ART consistently [39,40]. Most participants had maintained HIV treatment and viral suppression for years, with an average of 10 years since HIV diagnosis, reflecting long-term retention in care. While therapeutic communication with HCPs has been emphasized for individuals newly diagnosed with HIV [41], information provision alone may not enhance communication self-efficacy among those already familiar with their HCPs. Furthermore, although the informational content addressed diverse domains, such as sexual health care and social life, it did not improve self-efficacy in condom usage and health-related quality of life.

### Effectiveness of Self-Record Health Life in the App Group

The app group showed a significant improvement in self-efficacy for managing symptoms at 4 weeks compared with the link group. According to self-recorded symptoms on the ESSC app, a quarter of participants experienced symptoms that lasted from a minimum of one day up to 19 days. As symptom experiences are adverse signs of HIV progression and strongly related to the quality of life of people living with HIV, previous studies have developed mobile apps aimed at symptom management [42,43]. Similar to our study, a 4-week intervention using an app with educational content, self-assessment, and health tracking was shown to enhance symptom management knowledge [43]. Unlike that study, which included weekly group sessions on symptoms, our intervention did not provide customized interactive sessions. Instead, participants engaged in self-reflection through the “My Page” feature, where they could review their own health data. Interestingly, in another intervention study, web-based interactive sessions for patients did not significantly improve symptom management [44]. The authors explained that skill-learning processes may not effectively enhance self-efficacy in patients with high treatment adherence. Despite the absence of structured interactions, self-efficacy for managing symptoms improved, suggesting that self-recording alone may contribute to symptom self-management. Meanwhile, recording the mental diary of the ESSC app had no significant effect on managing depression or mood compared with the mobile link group. Rather, improvement was seen at 8 weeks in the link group that provided only psychological health care information. However, it is difficult to determine whether the mental diary had an adverse effect on mood management because the number of records was low between 4 and 8 weeks. Other apps specializing in symptom management [42,43], including psychological coping strategies for depression and anxiety severity, have been shown to help reduce psychological distress. User-customized psychiatric content beyond a mental diary is suggested for managing depressed moods.

### Challenges of App Improvement

Only one in 10 participants maintained their daily visits after completion of the intervention, during the voluntary use period between 4 and 8 weeks. In other app interventions requiring a query response for medication adherence, the response rate was 47.7% over 2 years [45]. The app provides weekly summary reports, and its most popular feature is the community message board, which allows users to share and interact with other users on social networks. The ESSC app has features for viewing summary reports at any time on “My Page,” but there is no community board for user interaction. This reflected the low need for peer communication among people living with HIV in the mHealth needs survey [11]. However, participants in this study gave feedback that they lacked motivation to continue using the app and expressed a desire for a community feature. By sharing experiences and common issues, people living with HIV can tap opportunities to perceive the usefulness of support groups and reduce stigma [46]. The community board should be operated in a safe space so that users do not encounter interpersonal conflicts [37], which will be refined in the next version of the ESSC app improvement. In addition, to maintain engagement with the app, participants responded that information should be updated regularly and interesting content should be provided to people living with HIV, such as a case of cure and the latest treatment development. HIV treatments have continued to develop in the evolution of ARTs, including injectable medication, and this progress has extended life expectancy and improved the quality of life [47]. Accordingly, it is natural that people living with HIV desire to stay up-to-date on the treatment, and the ESSC app should incorporate a dedicated news section with the latest information.

### Implications for mHealth Adoption

The link group, which accessed digital information via individual text messages without signing up for the app, showed significantly improved self-efficacy in managing depression or mood, fatigue, and getting support or help. While the mHealth app is an emerging technology for empowering patients’ self-management of chronic illnesses, it must also address potential risks to confidentiality and ensure patient autonomy [48]. Some users in this study perceived the ESSC app as an unnecessary device for good patients. The study by Jacomet et al [10] of people living with HIV and their physicians’ use patterns, benefits, and perceived barriers to different aspects of eHealth reported that 26% of people living with HIV and 43% of physicians were enthusiastic about eHealth, whereas 31% and 37%, respectively, expressed skepticism. To adopt mHealth for patients, multiple factors should be considered, including user characteristics, literacy, system utility, technology infrastructure, collaboration, and regulatory settings [49]. Diversifying the range of mHealth services available to patients can reduce barriers to accessing digital health resources and contribute to health equity. Link-based access can be proposed as an alternative mHealth strategy to bridge the gap between people living with HIV who are hesitant to use apps and those who use app in the context of digital information technology.

### Limitations

There are several limitations in interpreting the results. First, the inclusion criteria for smartphone users may not be representative of the generalized findings for people living with HIV. To ensure representativeness and health equity, further studies are needed to enhance community-based enrollment without limiting smartphone literacy. Second, although the final sample size exceeded the minimum required (n=60) based on a priori power analysis, it did not reach the target of 86 participants. While the final sample size was sufficient to detect moderate effect sizes, the reduced sample size may have limited the ability to identify smaller differences between groups. This limitation should be considered when interpreting the findings. Third, the 4-week intervention period may have been too short to fully assess the effectiveness and usability of the app. To observe sustained engagement patterns, an additional 4-week voluntary-use period was provided. However, voluntary use during the follow-up period was notably low. We designed a relatively short intervention period, as the primary aim of this study was to evaluate the impact of 2 types of fully non-face-to-face, information-driven mHealth delivery formats. To enhance usability and engagement for longer-term interventions, future versions of the app should incorporate additional features, such as user-interactive community boards, that go beyond information provision and self-recorded healthy living. To determine the practical usefulness of the ESSC app, it is necessary to evaluate its long-term effectiveness following future app improvements.

### Conclusions

The provision of integrated information using mobile devices improves HIV-SE among people living with HIV. Both mobile apps and link accessing in text messages are useful methods for providing non-face-to-face information. People living with HIV who have already been retained in care may not prefer to become online members of HIV-related apps, and mobile-based interventions should be applied according to individual preferences. While an informational mobile app that allows users to self-record and reflect on their healthy life improved self-efficacy for managing symptoms compared with link accessing in text messages, the app has challenges in maintaining long-term use. Usability findings suggest that up-to-date news sections and user-to-user community features are required for long-term engagement with informational mobile apps.

## Supplementary material

10.2196/60905 Checklist 1 CONSORT-EHEALTH V1.6 check list. CONSORT: Consolidated Standards of Reporting Trails.

10.2196/60905 Checklist 2 CONSORT 2010 checklist. CONSORT: Consolidated Standards of Reporting Trails.

## Abbreviations

ART: antiretroviral therapy
CD4+T: CD4+T lymphocyte
ESSC: Excellent Self-Supervised HIV Care
GEE: generalized estimating equation
HCP: health care provider
Health-ITUES: Health Information Technology Usability Evaluation Scale
HIV-SE: self-efficacy for HIV management
mHealth: mobile health
SF-HLS: Short-Form Health Literacy Scale
VAS: visual analogue scale
WHOQoL-HIV BREF: World Health Organization Quality of Life–HIV questionnaire
