# Supplementary material for: Providing 2 Types of mHealth Interventions to Support Self-Management Among People Living With HIV: Randomized Clinical Trial
Source: JMIR Mhealth Uhealth. 2025 May 29;13:e60905. doi: 10.2196/60905 (PMC12140505; doi:10.2196/60905)
Supplement: Checklist 1 [file mhealth-v13-e60905-s001.pdf]

CONSORT-EHEALTH (V 1.6.1) - Submission/Publication Form

The CONSORT-EHEALTH checklist is intended for authors of randomized trials evaluating web-based and Internet-based applications/interventions, including mobile interventions, electronic games (incl multiplayer games), social media, certain telehealth applications, and other interactive and/or networked electronic applications. Some of the items (e.g. all subitems under item 5 - description of the intervention) may also be applicable for other study designs.

The goal of the CONSORT EHEALTH checklist and guideline is to be
a) a guide for reporting for authors of RCTs,
b) to form a basis for appraisal of an ehealth trial (in terms of validity)

CONSORT-EHEALTH items/subitems are MANDATORY reporting items for studies published in the Journal of Medical Internet Research and other journals / scientific societies endorsing the checklist.

Items numbered 1., 2., 3., 4a., 4b etc are original CONSORT or CONSORT-NPT (non-pharmacologic treatment) items.
Items with Roman numerals (i., ii, iii, iv etc.) are CONSORT-EHEALTH extensions/clarifications.

As the CONSORT-EHEALTH checklist is still considered in a formative stage, we would ask that you also RATE ON A SCALE OF 1-5 how important/useful you feel each item is FOR THE PURPOSE OF THE CHECKLIST and reporting guideline (optional).

Mandatory reporting items are marked with a red *.
In the textboxes, either copy & paste the relevant sections from your manuscript into this form - please include any quotes from your manuscript in QUOTATION MARKS,
or answer directly by providing additional information not in the manuscript, or elaborating on why the item was not relevant for this study.

YOUR ANSWERS WILL BE PUBLISHED AS A SUPPLEMENTARY FILE TO YOUR PUBLICATION IN JMIR AND ARE CONSIDERED PART OF YOUR PUBLICATION (IF ACCEPTED).
Please fill in these questions diligently. Information will not be copyedited, so please use proper spelling and grammar, use correct capitalization, and avoid abbreviations.

DO NOT FORGET TO SAVE AS PDF _AND_ CLICK THE SUBMIT BUTTON SO YOUR ANSWERS ARE IN OUR DATABASE !!!

Citation Suggestion (if you append the pdf as Appendix we suggest to cite this paper in the caption):
Eysenbach G, CONSORT-EHEALTH Group
CONSORT-EHEALTH: Improving and Standardizing Evaluation Reports of Web-based and Mobile Health Interventions
J Med Internet Res 2011;13(4):e126

URL: http://www.jmir.org/2011/4/e126/
doi: 10.2196/jmir.1923
PMID: 22209829

laykim0618@gmail.com 계정 전환

비공개

초안 저장됨

* 표시는 필수 질문임

Your name *

First Last

Layoung Kim

Primary Affiliation (short), City, Country *

University of Toronto, Toronto, Canada

Mo-Im Kim Nursing Research Institute, College

Your e-mail address *

abc@gmail.com

laykim0618@gmail.com

Title of your manuscript *

Provide the (draft) title of your manuscript.

Given Two Types of mHealth Interventions to Support Self-Management among People Living with HIV: Randomized Clinical Trial

Name of your App/Software/Intervention *

If there is a short and a long/alternate name, write the short name first and add the long name in brackets.

ESSC app (Excellent Self Supervised HIV Care)

Evaluated Version (if any)

e.g. "V1", "Release 2017-03-01", "Version 2.0.27913"

ESSC V1

Language(s) *

What language is the intervention/app in? If multiple languages are available, separate by comma (e.g. "English, French")

Korean

URL of your Intervention Website or App

e.g. a direct link to the mobile app on app in appstore (itunes, Google Play), or URL of the website. If the intervention is a DVD or hardware, you can also link to an Amazon page.

https://play.google.com/store/apps/details?id=com.research.essg&hl=ko&gl=US

URL of an image/screenshot (optional)

N/A

Accessibility *

Can an enduser access the intervention presently?

- ○ access is free and open
- ◉ access only for special usergroups, not open
- ○ access is open to everyone, but requires payment/subscription/in-app purchases
- ○ app/intervention no longer accessible
- ○ 기타: ______________________

Primary Medical Indication/Disease/Condition *

e.g. "Stress", "Diabetes", or define the target group in brackets after the condition, e.g. "Autism (Parents of children with)", "Alzheimers (Informal Caregivers of)"

People living with HIV

Primary Outcomes measured in trial *

comma-separated list of primary outcomes reported in the trial

Self-efficacy for HIV management

Secondary/other outcomes

Are there any other outcomes the intervention is expected to affect?

Medication adherence, Self-efficacy about condom usage, Health-Related Quality of Life

!

Recommended "Dose" *

What do the instructions for users say on how often the app should be used?

- (selected) Approximately Daily
- Approximately Weekly
- Approximately Monthly
- Approximately Yearly
- "as needed"
- 기타: ____

Approx. Percentage of Users (starters) still using the app as recommended after 3 months *

- (selected) unknown / not evaluated
- 0-10%
- 11-20%
- 21-30%
- 31-40%
- 41-50%
- 51-60%
- 61-70%
- 71%-80%
- 81-90%
- 91-100%
- 기타: ____

Overall, was the app/intervention effective? *

- ( ) yes: all primary outcomes were significantly better in intervention group vs control
- (●) partly: SOME primary outcomes were significantly better in intervention group vs control
- ( ) no statistically significant difference between control and intervention
- ( ) potentially harmful: control was significantly better than intervention in one or more outcomes
- ( ) inconclusive: more research is needed
- ( ) 기타: ____________________

Article Preparation Status/Stage *

At which stage in your article preparation are you currently (at the time you fill in this form)

- ( ) not submitted yet - in early draft status
- (●) not submitted yet - in late draft status, just before submission
- ( ) submitted to a journal but not reviewed yet
- ( ) submitted to a journal and after receiving initial reviewer comments
- ( ) submitted to a journal and accepted, but not published yet
- ( ) published
- ( ) 기타: ____________________

Journal *

If you already know where you will submit this paper (or if it is already submitted), please provide the journal name (if it is not JMIR, provide the journal name under "other")

- ○ not submitted yet / unclear where I will submit this
- ○ Journal of Medical Internet Research (JMIR)
- ◉ JMIR mHealth and UHealth
- ○ JMIR Serious Games
- ○ JMIR Mental Health
- ○ JMIR Public Health
- ○ JMIR Formative Research
- ○ Other JMIR sister journal
- ○ 기타: ______

Is this a full powered effectiveness trial or a pilot/feasibility trial? *

- ◉ Pilot/feasibility
- ○ Fully powered

Manuscript tracking number *

If this is a JMIR submission, please provide the manuscript tracking number under "other" (The ms tracking number can be found in the submission acknowledgement email, or when you login as author in JMIR. If the paper is already published in JMIR, then the ms tracking number is the four-digit number at the end of the DOI, to be found at the bottom of each published article in JMIR)

- ◉ no ms number (yet) / not (yet) submitted to / published in JMIR
- ○ 기타: ______

TITLE AND ABSTRACT

1a) TITLE: Identification as a randomized trial in the title

1a) Does your paper address CONSORT item 1a? *

I.e does the title contain the phrase "Randomized Controlled Trial"? (if not, explain the reason under "other")

- (selected) yes
- 기타:

1a-i) Identify the mode of delivery in the title

Identify the mode of delivery. Preferably use “web-based” and/or “mobile” and/or “electronic game” in the title. Avoid ambiguous terms like “online”, “virtual”, “interactive”. Use “Internet-based” only if Intervention includes non-web-based Internet components (e.g. email), use “computer-based” or “electronic” only if offline products are used. Use “virtual” only in the context of “virtual reality” (3-D worlds). Use “online” only in the context of “online support groups”. Complement or substitute product names with broader terms for the class of products (such as “mobile” or “smart phone” instead of “iphone”), especially if the application runs on different platforms.

	1	2	3	4	5	
subitem not at all important	○	○	○	○	●	essential

선택해제

Does your paper address subitem 1a-i? *

Copy and paste relevant sections from manuscript title (include quotes in quotation marks "like this" to indicate direct quotes from your manuscript), or elaborate on this item by providing additional information not in the ms, or briefly explain why the item is not applicable/relevant for your study

Given "Two Types of mHealth Interventions" to Support Self-Management among People Living with HIV: Randomized Clinical Trial

1a-ii) Non-web-based components or important co-interventions in title

Mention non-web-based components or important co-interventions in title, if any (e.g., “with telephone support”).

	1	2	3	4	5	
subitem not at all important	◉	○	○	○	○	essential

선택해제

Does your paper address subitem 1a-ii?

Copy and paste relevant sections from manuscript title (include quotes in quotation marks "like this" to indicate direct quotes from your manuscript), or elaborate on this item by providing additional information not in the ms, or briefly explain why the item is not applicable/relevant for your study

Because we did not conduct any intervention without using the mobile app or mobile text message

1a-iii) Primary condition or target group in the title

Mention primary condition or target group in the title, if any (e.g., “for children with Type I Diabetes”) Example: A Web-based and Mobile Intervention with Telephone Support for Children with Type I Diabetes: Randomized Controlled Trial

	1	2	3	4	5	
subitem not at all important	○	○	○	○	◉	essential

선택해제

Does your paper address subitem 1a-iii? *

Copy and paste relevant sections from manuscript title (include quotes in quotation marks "like this" to indicate direct quotes from your manuscript), or elaborate on this item by providing additional information not in the ms, or briefly explain why the item is not applicable/relevant for your study

Given Two Types of mHealth Interventions to Support Self-Management among "People Living with HIV": Randomized Clinical Trial

1b) ABSTRACT: Structured summary of trial design, methods, results, and conclusions

NPT extension: Description of experimental treatment, comparator, care providers, centers, and blinding status.

1b-i) Key features/functionalities/components of the intervention and comparator in the METHODS section of the ABSTRACT

Mention key features/functionalities/components of the intervention and comparator in the abstract. If possible, also mention theories and principles used for designing the site. Keep in mind the needs of systematic reviewers and indexers by including important synonyms. (Note: Only report in the abstract what the main paper is reporting. If this information is missing from the main body of text, consider adding it)

	1	2	3	4	5	
subitem not at all important	○	○	○	○	◉	essential

선택해제

Does your paper address subitem 1b-i? *

Copy and paste relevant sections from the manuscript abstract (include quotes in quotation marks "like this" to indicate direct quotes from your manuscript), or elaborate on this item by providing additional information not in the ms, or briefly explain why the item is not applicable/relevant for your study

We compared the HIV self-management impact of two non-face-to-face interventions: "mobile link access information (Link group) and a mobile app to explore information and self-record health outcomes such as medication adherence, symptoms, mental score, and sexual safety (App group)".

1b-ii) Level of human involvement in the METHODS section of the ABSTRACT

Clarify the level of human involvement in the abstract, e.g., use phrases like “fully automated” vs. “therapist/nurse/care provider/physician-assisted” (mention number and expertise of providers involved, if any). (Note: Only report in the abstract what the main paper is reporting. If this information is missing from the main body of text, consider adding it)

	1	2	3	4	5	
subitem not at all important	◉	○	○	○	○	essential

선택해제

Does your paper address subitem 1b-ii?

Copy and paste relevant sections from the manuscript abstract (include quotes in quotation marks "like this" to indicate direct quotes from your manuscript), or elaborate on this item by providing additional information not in the ms, or briefly explain why the item is not applicable/relevant for your study

The informational mobile-based interventions were conducted fully non-face-to-face. mHealth could communicate with the healthcare provider if the user wishes.

1b-iii) Open vs. closed, web-based (self-assessment) vs. face-to-face assessments in the METHODS section of the ABSTRACT

Mention how participants were recruited (online vs. offline), e.g., from an open access website or from a clinic or a closed online user group (closed usergroup trial), and clarify if this was a purely web-based trial, or there were face-to-face components (as part of the intervention or for assessment). Clearly say if outcomes were self-assessed through questionnaires (as common in web-based trials). Note: In traditional offline trials, an open trial (open-label trial) is a type of clinical trial in which both the researchers and participants know which treatment is being administered. To avoid confusion, use “blinded” or “unblinded” to indicated the level of blinding instead of “open”, as “open” in web-based trials usually refers to “open access” (i.e. participants can self-enrol). (Note: Only report in the abstract what the main paper is reporting. If this information is missing from the main body of text, consider adding it)

	1	2	3	4	5	
subitem not at all important	○	○	○	○	◉	essential

선택해제

Does your paper address subitem 1b-iii?

Copy and paste relevant sections from the manuscript abstract (include quotes in quotation marks "like this" to indicate direct quotes from your manuscript), or elaborate on this item by providing additional information not in the ms, or briefly explain why the item is not applicable/relevant for your study

We compared HIV self-management impacts of two "non-face-to-face" interventions.~~~~
The primary outcome was "self-reported" self-efficacy for HIV management (HIV-SE) which consists of six domains

1b-iv) RESULTS section in abstract must contain use data

Report number of participants enrolled/assessed in each group, the use/uptake of the intervention (e.g., attrition/adherence metrics, use over time, number of logins etc.), in addition to primary/secondary outcomes. (Note: Only report in the abstract what the main paper is reporting. If this information is missing from the main body of text, consider adding it)

	1	2	3	4	5	
subitem not at all important	○	○	○	○	◉	essential

선택해제

Does your paper address subitem 1b-iv?

Copy and paste relevant sections from the manuscript abstract (include quotes in quotation marks "like this" to indicate direct quotes from your manuscript), or elaborate on this item by providing additional information not in the ms, or briefly explain why the item is not applicable/relevant for your study

A two-arm, prospective, randomized clinical trial was conducted, involving 83 PLWH aged ≥19, who were assigned to "the App group (n=42) or Link group (n=41)". The GEE analysis indicated that the App group had significant differential changes with a positive effect on managing symptoms at 4 weeks (β=0.635, p=.042) and a negative effect on managing depression/mood at 8 weeks (β=-0.824, p=.010) compared to the Link group. "Only 9.5% of participants in the App group maintained daily visits for a voluntary use period of 4 to 8 weeks".

1b-v) CONCLUSIONS/DISCUSSION in abstract for negative trials

Conclusions/Discussions in abstract for negative trials: Discuss the primary outcome - if the trial is negative (primary outcome not changed), and the intervention was not used, discuss whether negative results are attributable to lack of uptake and discuss reasons. (Note: Only report in the abstract what the main paper is reporting. If this information is missing from the main body of text, consider adding it)

	1	2	3	4	5	
subitem not at all important	○	○	○	○	◉	essential

선택해제

Does your paper address subitem 1b-v?

Copy and paste relevant sections from the manuscript abstract (include quotes in quotation marks "like this" to indicate direct quotes from your manuscript), or elaborate on this item by providing additional information not in the ms, or briefly explain why the item is not applicable/relevant for your study

Self-recording health outcomes and self-reflection through mobile apps influence symptom management; "however, further strategies are needed to ensure long-term app use. User-customized psychiatric content beyond mental health recordings has been suggested for managing depressed moods in mHealth interventions."

INTRODUCTION

2a) In INTRODUCTION: Scientific background and explanation of rationale

2a-i) Problem and the type of system/solution

Describe the problem and the type of system/solution that is object of the study: intended as stand-alone intervention vs. incorporated in broader health care program? Intended for a particular patient population? Goals of the intervention, e.g., being more cost-effective to other interventions, replace or complement other solutions? (Note: Details about the intervention are provided in “Methods” under 5)

	1	2	3	4	5	
subitem not at all important	○	○	○	○	◉	essential

선택해제

Does your paper address subitem 2a-i? *

Copy and paste relevant sections from the manuscript (include quotes in quotation marks "like this" to indicate direct quotes from your manuscript), or elaborate on this item by providing additional information not in the ms, or briefly explain why the item is not applicable/relevant for your study

Notably, it is challenging to find "mobile health (mHealth) diversity focused on the majority of PLWH who adhere to ART and are retained in care". In addition, studies have reported that the adoption and use of apps account for only a small proportion [7, 8]. Despite the expansion of digital health information, PLWHs desired evidence-based, trusted information in the mHealth requirement survey [9]. These findings suggest that "community-dwelling PLWHs" still require informational mHealth.

2a-ii) Scientific background, rationale: What is known about the (type of) system

Scientific background, rationale: What is known about the (type of) system that is the object of the study (be sure to discuss the use of similar systems for other conditions/diagnoses, if appropiate), motivation for the study, i.e. what are the reasons for and what is the context for this specific study, from which stakeholder viewpoint is the study performed, potential impact of findings [2]. Briefly justify the choice of the comparator.

	1	2	3	4	5	
subitem not at all important	○	○	○	○	●	essential

선택해제

Does your paper address subitem 2a-ii? *

Copy and paste relevant sections from the manuscript (include quotes in quotation marks "like this" to indicate direct quotes from your manuscript), or elaborate on this item by providing additional information not in the ms, or briefly explain why the item is not applicable/relevant for your study

"Considering that many PLWHs achieve ART adherence, integrated care has been emphasized to empower them to play a role in the HIV care continuum [10, 11]." "In this study, the information content comprised six domains—HIV management, ART care, psychological healthcare, sexual healthcare, health promotion, and family and social life—that could provide knowledge and information regarding integrated health services [9, 10]. Informational mHealth is designed to be provided using a mobile app or link accessing in text messages. Although downloading and keeping the app to improve self-efficacy for HIV management is a useful behavior for PLWH, it could pose a risk of individuals' HIV status being exposed to others. The coexistence of concealment and exposure [13] should be ensured and understood in the context of application."

2b) In INTRODUCTION: Specific objectives or hypotheses

Does your paper address CONSORT subitem 2b? *

Copy and paste relevant sections from the manuscript (include quotes in quotation marks "like this" to indicate direct quotes from your manuscript), or elaborate on this item by providing additional information not in the ms, or briefly explain why the item is not applicable/relevant for your study

"This study assesses the effectiveness of informational mHealth in improving self-efficacy for HIV management. We evaluate the effectiveness of the two mHealth interventions in the pre- post test and compare the impact of self-efficacy for HIV management between the mobile app and links."

METHODS

3a) Description of trial design (such as parallel, factorial) including allocation ratio

Does your paper address CONSORT subitem 3a? *

Copy and paste relevant sections from the manuscript (include quotes in quotation marks "like this" to indicate direct quotes from your manuscript), or elaborate on this item by providing additional information not in the ms, or briefly explain why the item is not applicable/relevant for your study

"This study was a two-arm randomized clinical trial." "Block randomization (block size of 4) was used to ensure 1:1 balanced enrollment. Those who generated the random number and block sequences (i.e., AABB, ABAB, BAAB, BABA, BBAA, ABBA) using Microsoft Excel and those who registered participants were different research staff. However, the research staff could not be blinded after the arm assignment to be involved in data control. Participants were unable to blind the assignment in the App-based trial. The research staff and participants did not know each other before and had never met."

3b) Important changes to methods after trial commencement (such as eligibility criteria), with reasons

Does your paper address CONSORT subitem 3b? *

Copy and paste relevant sections from the manuscript (include quotes in quotation marks "like this" to indicate direct quotes from your manuscript), or elaborate on this item by providing additional information not in the ms, or briefly explain why the item is not applicable/relevant for your study

There were no method changes after the start of the study.

3b-i) Bug fixes, Downtimes, Content Changes

Bug fixes, Downtimes, Content Changes: ehealth systems are often dynamic systems. A description of changes to methods therefore also includes important changes made on the intervention or comparator during the trial (e.g., major bug fixes or changes in the functionality or content) (5-iii) and other “unexpected events” that may have influenced study design such as staff changes, system failures/downtimes, etc. [2].

	1	2	3	4	5	
subitem not at all important	○	○	○	○	◉	essential

선택해제

Does your paper address subitem 3b-i?

Copy and paste relevant sections from the manuscript (include quotes in quotation marks "like this" to indicate direct quotes from your manuscript), or elaborate on this item by providing additional information not in the ms, or briefly explain why the item is not applicable/relevant for your study

There were no unexpected events or changes

4a) Eligibility criteria for participants

Does your paper address CONSORT subitem 4a? *

Copy and paste relevant sections from the manuscript (include quotes in quotation marks "like this" to indicate direct quotes from your manuscript), or elaborate on this item by providing additional information not in the ms, or briefly explain why the item is not applicable/relevant for your study

"The eligibility criteria were as follows: (1) PLWHs diagnosed with HIV, aged 19 and older, (2) individuals who owned and used a smartphone, and (3) those who had no difficulty reading and responding to the questionnaire via smartphone. Participants were excluded if they had a medical diagnosis that required immediate treatment and counseling, other than HIV infection."

4a-i) Computer / Internet literacy

Computer / Internet literacy is often an implicit "de facto" eligibility criterion - this should be explicitly clarified.

	1	2	3	4	5	
subitem not at all important	○	○	○	○	◉	essential

선택해제

Does your paper address subitem 4a-i?

Copy and paste relevant sections from the manuscript (include quotes in quotation marks "like this" to indicate direct quotes from your manuscript), or elaborate on this item by providing additional information not in the ms, or briefly explain why the item is not applicable/relevant for your study

"(3) those who had no difficulty reading and responding to the questionnaire via smartphone"

4a-ii) Open vs. closed, web-based vs. face-to-face assessments:

Open vs. closed, web-based vs. face-to-face assessments: Mention how participants were recruited (online vs. offline), e.g., from an open access website or from a clinic, and clarify if this was a purely web-based trial, or there were face-to-face components (as part of the intervention or for assessment), i.e., to what degree got the study team to know the participant. In online-only trials, clarify if participants were quasi-anonymous and whether having multiple identities was possible or whether technical or logistical measures (e.g., cookies, email confirmation, phone calls) were used to detect/prevent these.

	1	2	3	4	5	
subitem not at all important	○	○	○	○	◉	essential

선택해제

Does your paper address subitem 4a-ii? *

Copy and paste relevant sections from the manuscript (include quotes in quotation marks "like this" to indicate direct quotes from your manuscript), or elaborate on this item by providing additional information not in the ms, or briefly explain why the item is not applicable/relevant for your study

"The research staff and participants did not know each other before and had never met. All contacts from the research team were made via text messages or e-mail, and a phone number was provided for participants to call 24/7 if needed." "All interventions and assessments were implemented through non-face-to-face contact, maintaining the privacy and confidentiality of the participants. They participated anonymously and could stop participating at any time through non-response." "Participants were recruited using an online access method." "The poster and leaflets specified the study purpose and participation method, and included a QR code that could access the online baseline survey. The online survey was administered only after reading and clicking on the informed consent in the online site. Participants who completed the baseline survey were randomly assigned to the App or Link groups."

4a-iii) Information giving during recruitment

Information given during recruitment. Specify how participants were briefed for recruitment and in the informed consent procedures (e.g., publish the informed consent documentation as appendix, see also item X26), as this information may have an effect on user self-selection, user expectation and may also bias results.

	1	2	3	4	5	
subitem not at all important	○	○	○	○	◉	essential

선택해제

Does your paper address subitem 4a-iii?

Copy and paste relevant sections from the manuscript (include quotes in quotation marks "like this" to indicate direct quotes from your manuscript), or elaborate on this item by providing additional information not in the ms, or briefly explain why the item is not applicable/relevant for your study

"The poster and leaflets specified the study purpose and participation method, and included a QR code that could access the online baseline survey. The online survey was administered only after reading and clicking on the informed consent in the online site. Participants who completed the baseline survey were randomly assigned to the App or Link groups. The research staff sent the App group a text message explaining how to download the app from the App Store or Google Play. The app allowed participants to log in only after receiving approval from the research team. The Link group received a text message mentioning that they would receive a weekly text message including a link to access the information."

4b) Settings and locations where the data were collected

Does your paper address CONSORT subitem 4b? *

Copy and paste relevant sections from the manuscript (include quotes in quotation marks "like this" to indicate direct quotes from your manuscript), or elaborate on this item by providing additional information not in the ms, or briefly explain why the item is not applicable/relevant for your study

"Participants were recruited using an online access method. A prior study to identify the mobile app requirements of PLWHs was conducted at five hospitals, and about half of the participants expressed their willingness to participate in further mobile app interventions. A recruitment poster was emailed to 138 individuals who had left their email addresses and expressed interest in participating in the intervention study based on a previous service-needs assessment conducted in 2020–2021. In addition, 340 small leaflets that fit into a pocket were distributed to PLWH-visiting outpatients by medical staff at the two hospitals."

4b-i) Report if outcomes were (self-)assessed through online questionnaires

Clearly report if outcomes were (self-)assessed through online questionnaires (as common in web-based trials) or otherwise.

	1	2	3	4	5	
subitem not at all important	○	○	○	○	◉	essential

선택해제

Does your paper address subitem 4b-i? *

Copy and paste relevant sections from the manuscript (include quotes in quotation marks "like this" to indicate direct quotes from your manuscript), or elaborate on this item by providing additional information not in the ms, or briefly explain why the item is not applicable/relevant for your study

"The online survey was administered only after reading and clicking on the informed consent in the online site." "The period of the mHealth intervention was 4 weeks, and assessment points include baseline (T0), after completing the intervention at 4 weeks (T1), and follow-up 4 weeks post-intervention at 8 weeks (T2). All interventions and assessments were implemented through non-face-to-face contact, maintaining the privacy and confidentiality of the participants. They participated anonymously and could stop participating at any time through non-response."

4b-ii) Report how institutional affiliations are displayed

Report how institutional affiliations are displayed to potential participants [on ehealth media], as affiliations with prestigious hospitals or universities may affect volunteer rates, use, and reactions with regards to an intervention.(Not a required item – describe only if this may bias results)

	1	2	3	4	5	
subitem not at all important	(•)	( )	( )	( )	( )	essential

선택해제

Does your paper address subitem 4b-ii?

Copy and paste relevant sections from the manuscript (include quotes in quotation marks "like this" to indicate direct quotes from your manuscript), or elaborate on this item by providing additional information not in the ms, or briefly explain why the item is not applicable/relevant for your study

The researcher's affiliated university was indicated on participant recruitment posters or leaflets. We did not consider that this would bias the results.

5) The interventions for each group with sufficient details to allow replication, including how and when they were actually administered

5-i) Mention names, credential, affiliations of the developers, sponsors, and owners

Mention names, credential, affiliations of the developers, sponsors, and owners [6] (if authors/evaluators are owners or developer of the software, this needs to be declared in a “Conflict of interest” section or mentioned elsewhere in the manuscript).

	1	2	3	4	5	
subitem not at all important	( )	( )	( )	( )	(•)	essential

선택해제

Does your paper address subitem 5-i?

Copy and paste relevant sections from the manuscript (include quotes in quotation marks "like this" to indicate direct quotes from your manuscript), or elaborate on this item by providing additional information not in the ms, or briefly explain why the item is not applicable/relevant for your study

The app is a research product developed by the authors, and funding is specified in the manuscript. There is no conflict of interest. "This work was supported by the National Research Foundation of Korea grant funded by the South Korean Government (Ministry of Science and ICT) (no. 2020R1A2C101081713) (principal investigator [PI]: Gwang Suk Kim) "

5-ii) Describe the history/development process

Describe the history/development process of the application and previous formative evaluations (e.g., focus groups, usability testing), as these will have an impact on adoption/use rates and help with interpreting results.

	1	2	3	4	5	
subitem not at all important	○	○	○	○	◉	essential

선택해제

Does your paper address subitem 5-ii?

Copy and paste relevant sections from the manuscript (include quotes in quotation marks "like this" to indicate direct quotes from your manuscript), or elaborate on this item by providing additional information not in the ms, or briefly explain why the item is not applicable/relevant for your study

"This group used a mobile app to support their HIV self-management. The app, namely “ESSC (Excellent Self Supervised HIV Care)” was developed employing three iterative cycles of the Design Science Research framework [9]. Three cycles comprise relevance, derived from users' requirement need survey; rigor, organized based on the information–motivation–behavioral skills (IMB) model with validity being evaluated by experts; and design, which built artifacts including functions, contents, and features. Formative details of the development process are described in a previous study [9]."

5-iii) Revisions and updating

Revisions and updating. Clearly mention the date and/or version number of the application/intervention (and comparator, if applicable) evaluated, or describe whether the intervention underwent major changes during the evaluation process, or whether the development and/or content was “frozen” during the trial. Describe dynamic components such as news feeds or changing content which may have an impact on the replicability of the intervention (for unexpected events see item 3b).

	1	2	3	4	5	
subitem not at all important	○	○	○	○	◉	essential

선택해제

Does your paper address subitem 5-iii?

Copy and paste relevant sections from the manuscript (include quotes in quotation marks "like this" to indicate direct quotes from your manuscript), or elaborate on this item by providing additional information not in the ms, or briefly explain why the item is not applicable/relevant for your study

There were no changes or unexpected events after the start of the study.

5-iv) Quality assurance methods

Provide information on quality assurance methods to ensure accuracy and quality of information provided [1], if applicable.

	1	2	3	4	5	
subitem not at all important	○	○	○	○	◉	essential

선택해제

Does your paper address subitem 5-iv?

Copy and paste relevant sections from the manuscript (include quotes in quotation marks "like this" to indicate direct quotes from your manuscript), or elaborate on this item by providing additional information not in the ms, or briefly explain why the item is not applicable/relevant for your study

Formation details for the development process, including efforts to ensure quality, have been described in prior study

5-v) Ensure replicability by publishing the source code, and/or providing screenshots/screen-capture video, and/or providing flowcharts of the algorithms used

Ensure replicability by publishing the source code, and/or providing screenshots/screen-capture video, and/or providing flowcharts of the algorithms used. Replicability (i.e., other researchers should in principle be able to replicate the study) is a hallmark of scientific reporting.

	1	2	3	4	5	
subitem not at all important	○	○	○	○	◉	essential

선택해제

Does your paper address subitem 5-v?

Copy and paste relevant sections from the manuscript (include quotes in quotation marks "like this" to indicate direct quotes from your manuscript), or elaborate on this item by providing additional information not in the ms, or briefly explain why the item is not applicable/relevant for your study

"Figure 1. Screenshots of mobile link vs. mobile app"

5-vi) Digital preservation

Digital preservation: Provide the URL of the application, but as the intervention is likely to change or disappear over the course of the years; also make sure the intervention is archived (Internet Archive, webcitation.org, and/or publishing the source code or screenshots/videos alongside the article). As pages behind login screens cannot be archived, consider creating demo pages which are accessible without login.

	1	2	3	4	5	
subitem not at all important	○	○	◉	○	○	essential

선택해제

Does your paper address subitem 5-vi?

Copy and paste relevant sections from the manuscript (include quotes in quotation marks "like this" to indicate direct quotes from your manuscript), or elaborate on this item by providing additional information not in the ms, or briefly explain why the item is not applicable/relevant for your study

We are considering creating demo pages for the final mobile intervention. It is impossible now that the project is in progress.

5-vii) Access

Access: Describe how participants accessed the application, in what setting/context, if they had to pay (or were paid) or not, whether they had to be a member of specific group. If known, describe how participants obtained "access to the platform and Internet" [1]. To ensure access for editors/reviewers/readers, consider to provide a "backdoor" login account or demo mode for reviewers/readers to explore the application (also important for archiving purposes, see vi).

	1	2	3	4	5	
subitem not at all important	○	○	○	○	○	essential

Does your paper address subitem 5-vii? *

Copy and paste relevant sections from the manuscript (include quotes in quotation marks "like this" to indicate direct quotes from your manuscript), or elaborate on this item by providing additional information not in the ms, or briefly explain why the item is not applicable/relevant for your study

"The research staff sent the App group a text message explaining how to download the app from the App Store or Google Play. The app allowed participants to log in only after receiving approval from the research team. The Link group received a text message mentioning that they would receive a weekly text message including a link to access the information." The app uses Korean and an upgraded version of the project is currently in progress.

5-viii) Mode of delivery, features/functionalities/components of the intervention and comparator, and the theoretical framework

Describe mode of delivery, features/functionalities/components of the intervention and comparator, and the theoretical framework [6] used to design them (instructional strategy [1], behaviour change techniques, persuasive features, etc., see e.g., [7, 8] for terminology). This includes an in-depth description of the content (including where it is coming from and who developed it) [1],” whether [and how] it is tailored to individual circumstances and allows users to track their progress and receive feedback” [6]. This also includes a description of communication delivery channels and – if computer-mediated communication is a component – whether communication was synchronous or asynchronous [6]. It also includes information on presentation strategies [1], including page design principles, average amount of text on pages, presence of hyperlinks to other resources, etc. [1].

	1	2	3	4	5	
subitem not at all important	○	○	○	○	◉	essential

선택해제

Does your paper address subitem 5-viii? *

Copy and paste relevant sections from the manuscript (include quotes in quotation marks "like this" to indicate direct quotes from your manuscript), or elaborate on this item by providing additional information not in the ms, or briefly explain why the item is not applicable/relevant for your study

"App group-This group used a mobile app to support their HIV self-management. The app, namely "ESSC (Excellent Self Supervised HIV Care)" was developed employing three iterative cycles of the Design Science Research framework [9]. ~~~When users left various questions about information, personal concerns, and app features, a nurse with more than 10 years of experience in PLWH research responded within 24 hours."
Formation details for the features/functionalities have been described in the prior study.

5-ix) Describe use parameters

Describe use parameters (e.g., intended "doses" and optimal timing for use). Clarify what instructions or recommendations were given to the user, e.g., regarding timing, frequency, heaviness of use, if any, or was the intervention used ad libitum.

	1	2	3	4	5	
subitem not at all important	○	○	○	○	◉	essential

선택해제

Does your paper address subitem 5-ix?

Copy and paste relevant sections from the manuscript (include quotes in quotation marks "like this" to indicate direct quotes from your manuscript), or elaborate on this item by providing additional information not in the ms, or briefly explain why the item is not applicable/relevant for your study

"Both groups received text messages from the research team once a week to encourage their engagement with mHealth during the 4 weeks of intervention. The App group received a text message that encouraged the use of app functions, including the information screen, while the Link group was encouraged to read the information material. After completing the intervention period at 4 weeks (T1), the participants were informed that the mandatory use period ended and that they could use the app or link address autonomously for the next 4 weeks (T2). During the 4 weeks of voluntary use, the research team did not contact the participants."
For the mobile app, it is set to record daily medication. On the other hand, rather than setting the optimal timing for informational mHealth (app or link) use, we guided participants to use it for 4 weeks and encouraged use more than once a week.

5-x) Clarify the level of human involvement

Clarify the level of human involvement (care providers or health professionals, also technical assistance) in the e-intervention or as co-intervention (detail number and expertise of professionals involved, if any, as well as “type of assistance offered, the timing and frequency of the support, how it is initiated, and the medium by which the assistance is delivered”. It may be necessary to distinguish between the level of human involvement required for the trial, and the level of human involvement required for a routine application outside of a RCT setting (discuss under item 21 – generalizability).

	1	2	3	4	5	
subitem not at all important	○	○	○	◉	○	essential

선택해제

Does your paper address subitem 5-x?

Copy and paste relevant sections from the manuscript (include quotes in quotation marks "like this" to indicate direct quotes from your manuscript), or elaborate on this item by providing additional information not in the ms, or briefly explain why the item is not applicable/relevant for your study

"After completing the mental and sexual diaries, a pop-up message prompted users to communicate with the healthcare provider. When users left various questions about information, personal concerns, and app features, a nurse with more than 10 years of experience in PLWH research responded within 24 hours.", "When participants had questions about information or personal concerns, they could send text messages or e-mails to the research team."

5-xi) Report any prompts/reminders used

Report any prompts/reminders used: Clarify if there were prompts (letters, emails, phone calls, SMS) to use the application, what triggered them, frequency etc. It may be necessary to distinguish between the level of prompts/reminders required for the trial, and the level of prompts/reminders for a routine application outside of a RCT setting (discuss under item 21 – generalizability).

	1	2	3	4	5	
subitem not at all important	○	○	○	◉	○	essential

선택해제

Does your paper address subitem 5-xi? *

Copy and paste relevant sections from the manuscript (include quotes in quotation marks "like this" to indicate direct quotes from your manuscript), or elaborate on this item by providing additional information not in the ms, or briefly explain why the item is not applicable/relevant for your study

"Both groups received text messages from the research team once a week to encourage their engagement with mHealth during the 4 weeks of intervention. The App group received a text message that encouraged the use of app functions, including the information screen, while the Link group was encouraged to read the information material. After completing the intervention period at 4 weeks (T1), the participants were informed that the mandatory use period ended and that they could use the app or link address autonomously for the next 4 weeks (T2). During the 4 weeks of voluntary use, the research team did not contact the participants."
For the mobile app, it is set to record daily medication. On the other hand, rather than setting the optimal timing for informational mHealth (app or link) use, we guided participants to use it for 4 weeks and encouraged use more than once a week.

5-xii) Describe any co-interventions (incl. training/support)

Describe any co-interventions (incl. training/support): Clearly state any interventions that are provided in addition to the targeted eHealth intervention, as ehealth intervention may not be designed as stand-alone intervention. This includes training sessions and support [1]. It may be necessary to distinguish between the level of training required for the trial, and the level of training for a routine application outside of a RCT setting (discuss under item 21 – generalizability.

	1	2	3	4	5	
subitem not at all important	○	○	○	◉	○	essential

선택해제

Does your paper address subitem 5-xii? *

Copy and paste relevant sections from the manuscript (include quotes in quotation marks "like this" to indicate direct quotes from your manuscript), or elaborate on this item by providing additional information not in the ms, or briefly explain why the item is not applicable/relevant for your study

There were no interventions that were provided in addition to the targeted mHealth intervention.

6a) Completely defined pre-specified primary and secondary outcome measures, including how and when they were assessed

Does your paper address CONSORT subitem 6a? *

Copy and paste relevant sections from the manuscript (include quotes in quotation marks "like this" to indicate direct quotes from your manuscript), or elaborate on this item by providing additional information not in the ms, or briefly explain why the item is not applicable/relevant for your study

Measurement: Participants' basic information was measured at baseline (T0), and primary and secondary outcomes were assessed three times (T0, T1, and T2). The usability of the ESSC app was evaluated at 4 weeks (T1). All assessments were self-reported using an online questionnaire platform (Google Survey).
Primary outcome: Self-efficacy for HIV management
The Korean version of the Self-Efficacy for HIV Management Scale (HIV-SE) [12] was used. The 33 items comprised six domains: managing depression/mood (nine), medications (six), symptoms (five), and fatigue (four); communicating with the healthcare provider (four); and getting support/help (five). The participants rated confidence in their ability to perform specific behaviors on a 10-point Likert scale (1 = "not sure at all" to 10 = "totally sure"). The original scales developed by Shively et al. [17] were translated into Korean and validated the conceptual domains as a six-factor model. In previous studies [18-21], the six domains were used to identify specific subconcepts of self-efficacy and analyze the multivariate factors in predicting self-management. Cronbach's alpha for the Korean scale was .91 [12] and .93 in this study.
Secondary outcomes
Medication adherence was self-reported as a score measuring the rate of medication use in the past month. The scores ranged from 0 to 100 (0 = not taking the prescribed medications and 100 = taking all prescribed medications).
Self-efficacy regarding condom use was assessed using a single item on perceived behavioral control toward safe sexual intercourse. "If I have sex with my partner, I am confident that I always use a condom," the participants responded on a five-point Likert scale (1 = "not at all" to 5 ="very much").
Health-related quality of Life was assessed using the EuroQoL group's Visual Analogue Scale, which measures perceived health on a particular day on a scale ranging from 0 to 100 (0 = the worst health imaginable, 100 = the best health imaginable).

Usability test of the mobile app
Usability tests were conducted only for the App group. User records accumulated in the app were used to quantitatively assess usability. The Health Information Technology Usability Evaluation Scale (Health-ITUES) was employed to evaluate the quality of the ESSC application. Schnall et al. (2018) developed 20 items of Health-ITUES to evaluate the usability of a mobile app for symptom self-management of community-dwelling PLWHs on a five-point Likert scale (from 1 = "strongly disagree" to 5 = "strongly agree") [22]. The Korean version [23] was adapted for HIV disease self-management and includes four subscales: impact, perceived usefulness, ease of use, and user control. Finally, the participants were free to write about any problems they experienced while using the app or anything that needed to be corrected or supplemented. This qualitative feedback was also conducted in the link group.

6a-i) Online questionnaires: describe if they were validated for online use and apply CHERRIES items to describe how the questionnaires were designed/deployed

If outcomes were obtained through online questionnaires, describe if they were validated for online use and apply CHERRIES items to describe how the questionnaires were designed/deployed [9].

	1	2	3	4	5	
subitem not at all important	○	○	○	◉	○	essential

선택해제

Does your paper address subitem 6a-i?

Copy and paste relevant sections from manuscript text

"All assessments were self-reported using an online questionnaire platform (Google Survey)"

6a-ii) Describe whether and how “use” (including intensity of use/dosage) was defined/measured/monitored

Describe whether and how “use” (including intensity of use/dosage) was defined/measured/monitored (logins, logfile analysis, etc.). Use/adoption metrics are important process outcomes that should be reported in any ehealth trial.

	1	2	3	4	5	
subitem not at all important	○	○	○	◉	○	essential

선택해제

Does your paper address subitem 6a-ii?

Copy and paste relevant sections from manuscript text

"Usability test of the mobile app
Usability tests were conducted only for the App group. User records accumulated in the app were used to quantitatively assess usability. The Health Information Technology Usability Evaluation Scale (Health-ITUES) was employed to evaluate the quality of the ESSC application. Schnall et al. (2018) developed 20 items of Health-ITUES to evaluate the usability of a mobile app for symptom self-management of community-dwelling PLWHs on a five-point Likert scale (from 1 = “strongly disagree” to 5 = “strongly agree”) [22]. The Korean version [23] was adapted for HIV disease self-management and includes four subscales: impact, perceived usefulness, ease of use, and user control. Finally, the participants were free to write about any problems they experienced while using the app or anything that needed to be corrected or supplemented. This qualitative feedback was also conducted in the link group."

6a-iii) Describe whether, how, and when qualitative feedback from participants was obtained

Describe whether, how, and when qualitative feedback from participants was obtained (e.g., through emails, feedback forms, interviews, focus groups).

	1	2	3	4	5	
subitem not at all important	○	○	○	○	●	essential

선택해제

Does your paper address subitem 6a-iii?

Copy and paste relevant sections from manuscript text

"Usability test of the mobile app
Usability tests were conducted only for the App group. User records accumulated in the app were used to quantitatively assess usability. The Health Information Technology Usability Evaluation Scale (Health-ITUES) was employed to evaluate the quality of the ESSC application. Schnall et al. (2018) developed 20 items of Health-ITUES to evaluate the usability of a mobile app for symptom self-management of community-dwelling PLWHs on a five-point Likert scale (from 1 = “strongly disagree” to 5 = “strongly agree”) [22]. The Korean version [23] was adapted for HIV disease self-management and includes four subscales: impact, perceived usefulness, ease of use, and user control. Finally, the participants were free to write about any problems they experienced while using the app or anything that needed to be corrected or supplemented. This qualitative feedback was also conducted in the link group."

6b) Any changes to trial outcomes after the trial commenced, with reasons

Does your paper address CONSORT subitem 6b? *

Copy and paste relevant sections from the manuscript (include quotes in quotation marks "like this" to indicate direct quotes from your manuscript), or elaborate on this item by providing additional information not in the ms, or briefly explain why the item is not applicable/relevant for your study

There were no outcome changes after the start of the study.

7a) How sample size was determined

NPT: When applicable, details of whether and how the clustering by care provides or centers was addressed

7a-i) Describe whether and how expected attrition was taken into account when calculating the sample size

Describe whether and how expected attrition was taken into account when calculating the sample size.

	1	2	3	4	5	
subitem not at all important	○	○	○	◉	○	essential

선택해제

Does your paper address subitem 7a-i?

Copy and paste relevant sections from manuscript title (include quotes in quotation marks "like this" to indicate direct quotes from your manuscript), or elaborate on this item by providing additional information not in the ms, or briefly explain why the item is not applicable/relevant for your study

"The sample size was estimated using G*power 3.1.9.2. software. The required sample size was 30 per group when the effect size was .44; first type error, 5%; power .80 in the two groups, referring to a previous study on the effectiveness of a similarly-designed mobile program [14]. At least 86 participants were required, considering a dropout rate of approximately 30%."

7b) When applicable, explanation of any interim analyses and stopping guidelines

Does your paper address CONSORT subitem 7b? *

Copy and paste relevant sections from the manuscript (include quotes in quotation marks "like this" to indicate direct quotes from your manuscript), or elaborate on this item by providing additional information not in the ms, or briefly explain why the item is not applicable/relevant for your study

Interim analysis was not applicable in this study.

8a) Method used to generate the random allocation sequence

NPT: When applicable, how care providers were allocated to each trial group

Does your paper address CONSORT subitem 8a? *

Copy and paste relevant sections from the manuscript (include quotes in quotation marks "like this" to indicate direct quotes from your manuscript), or elaborate on this item by providing additional information not in the ms, or briefly explain why the item is not applicable/relevant for your study

"Block randomization (block size of 4) was used to ensure 1:1 balanced enrollment. Those who generated the random number and block sequences (i.e., AABB, ABAB, BAAB, BABA, BBAA, ABBA) using Microsoft Excel and those who registered participants were different research staff. However, the research staff could not be blinded after the arm assignment to be involved in data control. Participants were unable to blind the assignment in the App-based trial. The research staff and participants did not know each other before and had never met."

8b) Type of randomisation; details of any restriction (such as blocking and block size)

Does your paper address CONSORT subitem 8b? *

Copy and paste relevant sections from the manuscript (include quotes in quotation marks "like this" to indicate direct quotes from your manuscript), or elaborate on this item by providing additional information not in the ms, or briefly explain why the item is not applicable/relevant for your study

"Block randomization (block size of 4) was used to ensure 1:1 balanced enrollment. Those who generated the random number and block sequences (i.e., AABB, ABAB, BAAB, BABA, BBAA, ABBA) using Microsoft Excel and those who registered participants were different research staff. However, the research staff could not be blinded after the arm assignment to be involved in data control. Participants were unable to blind the assignment in the App-based trial. The research staff and participants did not know each other before and had never met."

9) Mechanism used to implement the random allocation sequence (such as sequentially numbered containers), describing any steps taken to conceal the sequence until interventions were assigned

Does your paper address CONSORT subitem 9? *

Copy and paste relevant sections from the manuscript (include quotes in quotation marks "like this" to indicate direct quotes from your manuscript), or elaborate on this item by providing additional information not in the ms, or briefly explain why the item is not applicable/relevant for your study

"Block randomization (block size of 4) was used to ensure 1:1 balanced enrollment. Those who generated the random number and block sequences (i.e., AABB, ABAB, BAAB, BABA, BBAA, ABBA) using Microsoft Excel and those who registered participants were different research staff. However, the research staff could not be blinded after the arm assignment to be involved in data control. Participants were unable to blind the assignment in the App-based trial. The research staff and participants did not know each other before and had never met."

10) Who generated the random allocation sequence, who enrolled participants, and who assigned participants to interventions

Does your paper address CONSORT subitem 10? *

Copy and paste relevant sections from the manuscript (include quotes in quotation marks "like this" to indicate direct quotes from your manuscript), or elaborate on this item by providing additional information not in the ms, or briefly explain why the item is not applicable/relevant for your study

"Those who generated the random number and block sequences (i.e., AABB, ABAB, BAAB, BABA, BBAA, ABBA) using Microsoft Excel and those who registered participants were different research staff. However, the research staff could not be blinded after the arm assignment to be involved in data control. Participants were unable to blind the assignment in the App-based trial. The research staff and participants did not know each other before and had never met."

11a) If done, who was blinded after assignment to interventions (for example, participants, care providers, those assessing outcomes) and how

NPT: Whether or not administering co-interventions were blinded to group assignment

11a-i) Specify who was blinded, and who wasn't

Specify who was blinded, and who wasn't. Usually, in web-based trials it is not possible to blind the participants [1, 3] (this should be clearly acknowledged), but it may be possible to blind outcome assessors, those doing data analysis or those administering co-interventions (if any).

	1	2	3	4	5	
subitem not at all important	○	○	○	◉	○	essential

선택해제

Does your paper address subitem 11a-i? *

Copy and paste relevant sections from the manuscript (include quotes in quotation marks "like this" to indicate direct quotes from your manuscript), or elaborate on this item by providing additional information not in the ms, or briefly explain why the item is not applicable/relevant for your study

"However, the research staff could not be blinded after the arm assignment to be involved in data control. Participants were unable to blind the assignment in the App-based trial. The research staff and participants did not know each other before and had never met. All contacts from the research team were made via text messages or e-mail, and a phone number was provided for participants to call 24/7 if needed.", "All interventions and assessments were implemented through non-face-to-face contact, maintaining the privacy and confidentiality of the participants. They participated anonymously and could stop participating at any time through non-response."

11a-ii) Discuss e.g., whether participants knew which intervention was the "intervention of interest" and which one was the "comparator"

Informed consent procedures (4a-ii) can create biases and certain expectations - discuss e.g., whether participants knew which intervention was the "intervention of interest" and which one was the "comparator".

	1	2	3	4	5	
subitem not at all important	○	○	○	◉	○	essential

선택해제

Does your paper address subitem 11a-ii?

Copy and paste relevant sections from the manuscript (include quotes in quotation marks "like this" to indicate direct quotes from your manuscript), or elaborate on this item by providing additional information not in the ms, or briefly explain why the item is not applicable/relevant for your study

"Although downloading and keeping the app to improve self-efficacy for HIV management is a useful behavior for PLWH, it could pose a risk of individuals' HIV status being exposed to others. The coexistence of concealment and exposure [13] should be ensured and understood in the context of application.", "While they had suitable conditions to participate in online support groups, only a quarter of PLWHs had experienced participating in self-help groups. In previous studies regarding online-based relationships, PLWHs noted difficulties in maintaining relationship, expressed concerns about exposing others to HIV infection, and information inaccuracy [34]", "Implications for mHealth adoption
The Link group, who could access digital information from individual text messages without signing up for the app, showed significantly improved self-efficacy in managing depression/mood, fatigue, and getting support/help. While the mHealth app is an emerging technology for empowering patients' self-management of chronic illnesses, it must also address potential risks to confidentiality and ensure patient autonomy [44]. Some users in this study perceived the ESSC app as an unnecessary device for good patients. Jacomet et al.'s study of PLWHs and their physicians' use patterns, benefits, and perceived barriers to different aspects of eHealth reported that 26% of PLWHs and 43% of physicians were enthusiastic about eHealth, while 31% and 37% were skeptical [8]. To adopt mHealth for patients, multiple factors including user characteristics, literacy, system utility, technology infrastructure, collaboration, and regulatory settings should be considered [45]. Diversifying the mHealth services available to patients can reduce barriers to accessing digital health resources and contribute to health equity. Link access can be proposed as a method of mHealth to bridge the gap between PLWHs who resist the use of apps and those who use apps in the context of digital information technology."

11b) If relevant, description of the similarity of interventions

(this item is usually not relevant for ehealth trials as it refers to similarity of a placebo or sham intervention to a active medication/intervention)

Does your paper address CONSORT subitem 11b? *

Copy and paste relevant sections from the manuscript (include quotes in quotation marks "like this" to indicate direct quotes from your manuscript), or elaborate on this item by providing additional information not in the ms, or briefly explain why the item is not applicable/relevant for your study

"We compared the Link group which received text messages of URL links for accessing information with the App group which used a mobile app for exploring information and self-recording health outcomes (Figure 1). The two interventional arms were implemented using participants' smartphones. The contents of the information provided were the same in both groups; however, the delivery methods were different. The Link group clicked on the URL address of the weekly received text messages, and the App group logged into the app to view the content posted at all times.", "While the mobile link provided only information, the mobile app had functions to self-record and view their recorded health life."

12a) Statistical methods used to compare groups for primary and secondary outcomes

NPT: When applicable, details of whether and how the clustering by care providers or centers was addressed

Does your paper address CONSORT subitem 12a? *

Copy and paste relevant sections from the manuscript (include quotes in quotation marks "like this" to indicate direct quotes from your manuscript), or elaborate on this item by providing additional information not in the ms, or briefly explain why the item is not applicable/relevant for your study

"Basic information variables at baseline are presented by the groups using descriptive analysis (counts with percentages or mean values with standard deviations). Changes in primary and secondary outcome variables between baseline and post-intervention were analyzed using paired two-sample t-tests, which examined the differences in the mean values of outcomes by group during the intervention. The primary outcome variables were compared between the App and Link groups using a generalized estimating equation (GEE). It is an appropriate statistical method that analyzes repeated measures data reflecting the interaction between group and time and performs intention-to-treat analysis. To compare the effectiveness of group differences across time, GEE analysis was performed, including the results of the three assessment points (T0, T1, and T2)."

12a-i) Imputation techniques to deal with attrition / missing values

Imputation techniques to deal with attrition / missing values: Not all participants will use the intervention/comparator as intended and attrition is typically high in ehealth trials. Specify how participants who did not use the application or dropped out from the trial were treated in the statistical analysis (a complete case analysis is strongly discouraged, and simple imputation techniques such as LOCF may also be problematic [4]).

	1	2	3	4	5	
subitem not at all important	○	○	○	◉	○	essential

선택해제

Does your paper address subitem 12a-i? *

Copy and paste relevant sections from the manuscript (include quotes in quotation marks "like this" to indicate direct quotes from your manuscript), or elaborate on this item by providing additional information not in the ms, or briefly explain why the item is not applicable/relevant for your study

"According to the statistical method, the GEE analysis included all the data of the withdrawn participants, but the paired t-test could not include withdrawn participants."

12b) Methods for additional analyses, such as subgroup analyses and adjusted analyses

Does your paper address CONSORT subitem 12b? *

Copy and paste relevant sections from the manuscript (include quotes in quotation marks "like this" to indicate direct quotes from your manuscript), or elaborate on this item by providing additional information not in the ms, or briefly explain why the item is not applicable/relevant for your study

"Here, the GEE analysis was adjusted for three covariates: number of years after HIV diagnosis, psychological domain of the WHOQoL-HIV BREF, and SF-HLS. Previous studies have shown that the HIV self-management ability of PLWHs was affected by the duration of HIV diagnosis, psychological status, and literacy [29-32]. In addition, health literacy scores were significantly different between the two groups in the homogeneity test of basic information using the chi-square test or independent t-test. Therefore, the research team acknowledged that these three covariates should be included in the evaluation of HIV-SE effectiveness."

X26) REB/IRB Approval and Ethical Considerations [recommended as subheading under "Methods"] (not a CONSORT item)

X26-i) Comment on ethics committee approval

	1	2	3	4	5	
subitem not at all important	○	○	○	○	◉	essential

선택해제

Does your paper address subitem X26-i?

Copy and paste relevant sections from the manuscript (include quotes in quotation marks "like this" to indicate direct quotes from your manuscript), or elaborate on this item by providing additional information not in the ms, or briefly explain why the item is not applicable/relevant for your study

"Ethical Considerations-
This study was approved by the Institutional Review Board of the first author's affiliation (4-2022-1192) and the protocol was registered with the Clinical Research Information Service (KCT0008014)"

x26-ii) Outline informed consent procedures

Outline informed consent procedures e.g., if consent was obtained offline or online (how? Checkbox, etc.?), and what information was provided (see 4a-ii). See [6] for some items to be included in informed consent documents.

	1	2	3	4	5	
subitem not at all important	○	○	○	○	◉	essential

선택해제

Does your paper address subitem X26-ii?

Copy and paste relevant sections from the manuscript (include quotes in quotation marks "like this" to indicate direct quotes from your manuscript), or elaborate on this item by providing additional information not in the ms, or briefly explain why the item is not applicable/relevant for your study

"The online survey was administered only after reading and clicking on the informed consent in the online site."

X26-iii) Safety and security procedures

Safety and security procedures, incl. privacy considerations, and any steps taken to reduce the likelihood or detection of harm (e.g., education and training, availability of a hotline)

	1	2	3	4	5	
subitem not at all important	○	○	○	○	◉	essential

선택해제

Does your paper address subitem X26-iii?

Copy and paste relevant sections from the manuscript (include quotes in quotation marks "like this" to indicate direct quotes from your manuscript), or elaborate on this item by providing additional information not in the ms, or briefly explain why the item is not applicable/relevant for your study

"All interventions and assessments were implemented through non-face-to-face contact, maintaining the privacy and confidentiality of the participants. They participated anonymously and could stop participating at any time through non-response."

RESULTS

13a) For each group, the numbers of participants who were randomly assigned, received intended treatment, and were analysed for the primary outcome

NPT: The number of care providers or centers performing the intervention in each group and the number of patients treated by each care provider in each center

Does your paper address CONSORT subitem 13a? *

Copy and paste relevant sections from the manuscript (include quotes in quotation marks "like this" to indicate direct quotes from your manuscript), or elaborate on this item by providing additional information not in the ms, or briefly explain why the item is not applicable/relevant for your study

"Figure 2 illustrates a flow diagram for trial recruitment. Overall, 87 individuals accessed online sites to participate. After responding to the baseline survey, four PLWHs were excluded as they met the exclusion criteria: had a psychiatric diagnosis (i.e., bipolar disorder or depression) with symptoms, and were under treatment. The remaining 83 PLWHs were assigned to the two groups, of which 73 completed the 8-week follow-up. When required to self-report assessments after completing the intervention at 4 weeks, 10 (12%) participants did not respond to the research team contact."

13b) For each group, losses and exclusions after randomisation, together with reasons

Does your paper address CONSORT subitem 13b? (NOTE: Preferably, this is shown in a CONSORT flow diagram) *

Copy and paste relevant sections from the manuscript (include quotes in quotation marks "like this" to indicate direct quotes from your manuscript), or elaborate on this item by providing additional information not in the ms, or briefly explain why the item is not applicable/relevant for your study

"Figure 2 illustrates a flow diagram for trial recruitment. Overall, 87 individuals accessed online sites to participate. After responding to the baseline survey, four PLWHs were excluded as they met the exclusion criteria: had a psychiatric diagnosis (i.e., bipolar disorder or depression) with symptoms, and were under treatment. The remaining 83 PLWHs were assigned to the two groups, of which 73 completed the 8-week follow-up. When required to self-report assessments after completing the intervention at 4 weeks, 10 (12%) participants did not respond to the research team contact."

13b-i) Attrition diagram

Strongly recommended: An attrition diagram (e.g., proportion of participants still logging in or using the intervention/comparator in each group plotted over time, similar to a survival curve) or other figures or tables demonstrating usage/dose/engagement.

	1	2	3	4	5	
subitem not at all important	○	○	○	○	◉	essential

선택해제

Does your paper address subitem 13b-i?

Copy and paste relevant sections from the manuscript or cite the figure number if applicable (include quotes in quotation marks "like this" to indicate direct quotes from your manuscript), or elaborate on this item by providing additional information not in the ms, or briefly explain why the item is not applicable/relevant for your study

"Regarding the quantitative usability of the ESSC app during the 4-week intervention period, 42.9% of App group participants completed the in-app logs fully, with medication adherence at 57.5%, mental diary at 54.8%, sexual diary at 64.3%, and treatment retention at 97.6% (Table 4). Only 9.5% maintained daily medication records from the completion of intervention to the voluntary use period of 4-8 weeks. The periods of app use per participant ID are presented in Figure 3. Individuals who recorded daily medication adherence tended to record weekly mental and sexual diaries."

14a) Dates defining the periods of recruitment and follow-up

Does your paper address CONSORT subitem 14a? *

Copy and paste relevant sections from the manuscript (include quotes in quotation marks "like this" to indicate direct quotes from your manuscript), or elaborate on this item by providing additional information not in the ms, or briefly explain why the item is not applicable/relevant for your study

"The period of the mHealth intervention was 4 weeks, and assessment points include baseline (T0), after completing the intervention at 4 weeks (T1), and follow-up 4 weeks post-intervention at 8 weeks (T2).", "After completing the intervention period at 4 weeks (T1), the participants were informed that the mandatory use period ended and that they could use the app or link address autonomously for the next 4 weeks (T2). During the 4 weeks of voluntary use, the research team did not contact the participants.", "Regarding the quantitative usability of the ESSC app during the 4-week intervention period, 42.9% of App group participants completed the in-app logs fully, with medication adherence at 57.5%, mental diary at 54.8%, sexual diary at 64.3%, and treatment retention at 97.6% (Table 4). Only 9.5% maintained daily medication records from the completion of intervention to the voluntary use period of 4-8 weeks. The periods of app use per participant ID are presented in Figure 3."

14a-i) Indicate if critical “secular events” fell into the study period

Indicate if critical “secular events” fell into the study period, e.g., significant changes in Internet resources available or “changes in computer hardware or Internet delivery resources”

	1	2	3	4	5	
subitem not at all important	●	○	○	○	○	essential

선택해제

Does your paper address subitem 14a-i?

Copy and paste relevant sections from the manuscript (include quotes in quotation marks "like this" to indicate direct quotes from your manuscript), or elaborate on this item by providing additional information not in the ms, or briefly explain why the item is not applicable/relevant for your study

secular events did not applicable to this study

14b) Why the trial ended or was stopped (early)

Does your paper address CONSORT subitem 14b? *

Copy and paste relevant sections from the manuscript (include quotes in quotation marks "like this" to indicate direct quotes from your manuscript), or elaborate on this item by providing additional information not in the ms, or briefly explain why the item is not applicable/relevant for your study

Stopped events were not applicable to this study

15) A table showing baseline demographic and clinical characteristics for each group

NPT: When applicable, a description of care providers (case volume, qualification, expertise, etc.) and centers (volume) in each group

Does your paper address CONSORT subitem 15? *

Copy and paste relevant sections from the manuscript (include quotes in quotation marks "like this" to indicate direct quotes from your manuscript), or elaborate on this item by providing additional information not in the ms, or briefly explain why the item is not applicable/relevant for your study

Table 1. Baseline characteristics of the study participants

15-i) Report demographics associated with digital divide issues

In ehealth trials it is particularly important to report demographics associated with digital divide issues, such as age, education, gender, social-economic status, computer/Internet/ehealth literacy of the participants, if known.

	1	2	3	4	5	
subitem not at all important	○	○	○	○	◉	essential

선택해제

Does your paper address subitem 15-i? *

Copy and paste relevant sections from the manuscript (include quotes in quotation marks "like this" to indicate direct quotes from your manuscript), or elaborate on this item by providing additional information not in the ms, or briefly explain why the item is not applicable/relevant for your study

"In addition, health literacy scores were significantly different between the two groups in the homogeneity test of basic information using the chi-square test or independent t-test. Therefore, the research team acknowledged that these three covariates should be included in the evaluation of HIV-SE effectiveness.", "The results of the homogeneity test showed that the SF-HLS scores (t= 2.667, p= .009) were significantly different between the two groups, while the other variables showed no significant differences."

16) For each group, number of participants (denominator) included in each analysis and whether the analysis was by original assigned groups

16-i) Report multiple “denominators” and provide definitions

Report multiple “denominators” and provide definitions: Report N’s (and effect sizes) “across a range of study participation [and use] thresholds” [1], e.g., N exposed, N consented, N used more than x times, N used more than y weeks, N participants “used” the intervention/comparator at specific pre-defined time points of interest (in absolute and relative numbers per group). Always clearly define “use” of the intervention.

	1	2	3	4	5	
subitem not at all important	○	○	○	○	◉	essential

선택해제

Does your paper address subitem 16-i? *

Copy and paste relevant sections from the manuscript (include quotes in quotation marks "like this" to indicate direct quotes from your manuscript), or elaborate on this item by providing additional information not in the ms, or briefly explain why the item is not applicable/relevant for your study

"Table 4. Number of users and records of mobile app group during the intervention period (n=42)", "Figure 3. Graph of the app usage by mobile app participant ID over 8 weeks"

16-ii) Primary analysis should be intent-to-treat

Primary analysis should be intent-to-treat, secondary analyses could include comparing only “users”, with the appropriate caveats that this is no longer a randomized sample (see 18-i).

	1	2	3	4	5	
subitem not at all important	○	○	○	○	◉	essential

선택해제

Does your paper address subitem 16-ii?

Copy and paste relevant sections from the manuscript (include quotes in quotation marks "like this" to indicate direct quotes from your manuscript), or elaborate on this item by providing additional information not in the ms, or briefly explain why the item is not applicable/relevant for your study

"The primary outcome variables were compared between the App and Link groups using a generalized estimating equation (GEE). It is an appropriate statistical method that analyzes repeated measures data reflecting the interaction between group and time and performs intention-to-treat analysis.", "According to the statistical method, the GEE analysis included all the data of the withdrawn participants, but the paired t-test could not include withdrawn participants."

17a) For each primary and secondary outcome, results for each group, and the estimated effect size and its precision (such as 95% confidence interval)

Does your paper address CONSORT subitem 17a? *

Copy and paste relevant sections from the manuscript (include quotes in quotation marks "like this" to indicate direct quotes from your manuscript), or elaborate on this item by providing additional information not in the ms, or briefly explain why the item is not applicable/relevant for your study

"Table 2. Differences in the mean of outcomes during the intervention period", "Table 3. Comparison of self-efficacy for HIV self-management between the mobile app and link group"

17a-i) Presentation of process outcomes such as metrics of use and intensity of use

In addition to primary/secondary (clinical) outcomes, the presentation of process outcomes such as metrics of use and intensity of use (dose, exposure) and their operational definitions is critical. This does not only refer to metrics of attrition (13-b) (often a binary variable), but also to more continuous exposure metrics such as “average session length”. These must be accompanied by a technical description how a metric like a “session” is defined (e.g., timeout after idle time) [1] (report under item 6a).

	1	2	3	4	5	
subitem not at all important	○	○	○	○	◉	essential

선택해제

Does your paper address subitem 17a-i?

Copy and paste relevant sections from the manuscript (include quotes in quotation marks "like this" to indicate direct quotes from your manuscript), or elaborate on this item by providing additional information not in the ms, or briefly explain why the item is not applicable/relevant for your study

"Table 4. Number of users and records of mobile app group during the intervention period (n=42)", "Figure 3. Graph of the app usage by mobile app participant ID over 8 weeks"

17b) For binary outcomes, presentation of both absolute and relative effect sizes is recommended

Does your paper address CONSORT subitem 17b? *

Copy and paste relevant sections from the manuscript (include quotes in quotation marks "like this" to indicate direct quotes from your manuscript), or elaborate on this item by providing additional information not in the ms, or briefly explain why the item is not applicable/relevant for your study

Binary outcomes did not apply to this study.

18) Results of any other analyses performed, including subgroup analyses and adjusted analyses, distinguishing pre-specified from exploratory

Does your paper address CONSORT subitem 18? *

Copy and paste relevant sections from the manuscript (include quotes in quotation marks "like this" to indicate direct quotes from your manuscript), or elaborate on this item by providing additional information not in the ms, or briefly explain why the item is not applicable/relevant for your study

Subgroup analyses did not apply to this study.

18-i) Subgroup analysis of comparing only users

A subgroup analysis of comparing only users is not uncommon in ehealth trials, but if done, it must be stressed that this is a self-selected sample and no longer an unbiased sample from a randomized trial (see 16-iii).

	1	2	3	4	5	
subitem not at all important	(●)	○	○	○	○	essential

선택해제

Does your paper address subitem 18-i?

Copy and paste relevant sections from the manuscript (include quotes in quotation marks "like this" to indicate direct quotes from your manuscript), or elaborate on this item by providing additional information not in the ms, or briefly explain why the item is not applicable/relevant for your study

Subgroup analyses did not apply to this study.

19) All important harms or unintended effects in each group
(for specific guidance see CONSORT for harms)

Does your paper address CONSORT subitem 19? *

Copy and paste relevant sections from the manuscript (include quotes in quotation marks "like this" to indicate direct quotes from your manuscript), or elaborate on this item by providing additional information not in the ms, or briefly explain why the item is not applicable/relevant for your study

There was no harm or unintended effects in each group

19-i) Include privacy breaches, technical problems

Include privacy breaches, technical problems. This does not only include physical “harm” to participants, but also incidents such as perceived or real privacy breaches [1], technical problems, and other unexpected/unintended incidents. “Unintended effects” also includes unintended positive effects [2].

	1	2	3	4	5	
subitem not at all important	(●)	( )	( )	( )	( )	essential

선택해제

Does your paper address subitem 19-i?

Copy and paste relevant sections from the manuscript (include quotes in quotation marks "like this" to indicate direct quotes from your manuscript), or elaborate on this item by providing additional information not in the ms, or briefly explain why the item is not applicable/relevant for your study

There was no privacy breaches, technical problems in each group

19-ii) Include qualitative feedback from participants or observations from staff/researchers

Include qualitative feedback from participants or observations from staff/researchers, if available, on strengths and shortcomings of the application, especially if they point to unintended/unexpected effects or uses. This includes (if available) reasons for why people did or did not use the application as intended by the developers.

	1	2	3	4	5	
subitem not at all important	○	○	○	○	◉	essential

선택해제

Does your paper address subitem 19-ii?

Copy and paste relevant sections from the manuscript (include quotes in quotation marks "like this" to indicate direct quotes from your manuscript), or elaborate on this item by providing additional information not in the ms, or briefly explain why the item is not applicable/relevant for your study

"Qualitative feedback regarding the ESSC app or the link is shown in Textbox 1. Participants believed that the information was easy to understand and important; they wanted to regularly receive updated information such as up-to-date treatment (e.g., long-acting injectable HIV antiretroviral therapy)."

DISCUSSION

22) Interpretation consistent with results, balancing benefits and harms, and considering other relevant evidence

NPT: In addition, take into account the choice of the comparator, lack of or partial blinding, and unequal expertise of care providers or centers in each group

22-i) Restate study questions and summarize the answers suggested by the data, starting with primary outcomes and process outcomes (use)

Restate study questions and summarize the answers suggested by the data, starting with primary outcomes and process outcomes (use).

	1	2	3	4	5	
subitem not at all important	○	○	○	○	◉	essential

선택해제

Does your paper address subitem 22-i? *

Copy and paste relevant sections from the manuscript (include quotes in quotation marks "like this" to indicate direct quotes from your manuscript), or elaborate on this item by providing additional information not in the ms, or briefly explain why the item is not applicable/relevant for your study

"Principal Results
This study is the first trial to conduct fully non-face-to-face digital interventions for community-dwelling PLWHs in Korea. Digital interventions for PLWHs have been designed to improve access to health information resources that are limited to hospital settings. Two-arm interventions were performed using a mobile app or link in text messages, which are popular implementation methods for mHealth. Comprehensive information that helps PLWHs self-manage their HIV disease and health life was provided by logging into the app or accessing links in the text messages. While the mobile link provided only information, the mobile app had functions to self-record and view their recorded health life. After 4 weeks, the total score of self-efficacy for HIV management increased from the baseline in both groups, and a statistically significant improvement was found in the app group. When the effectiveness of both interventions was compared using GEE, there was no significant differential change over 8 weeks in the total self-efficacy score. However, when comparing domain scores to the Link group, the App group showed significant differential changes, with a positive effect on managing symptoms at 4 weeks and a negative effect on managing depression/mood at 8 weeks. Our findings suggest that the information-driven mobile app helps improve self-efficacy for HIV disease management and that the function of self-recording health life has benefits for symptom management."

22-ii) Highlight unanswered new questions, suggest future research

Highlight unanswered new questions, suggest future research.

	1	2	3	4	5	
subitem not at all important	○	○	○	○	◉	essential

선택해제

Does your paper address subitem 22-ii?

Copy and paste relevant sections from the manuscript (include quotes in quotation marks "like this" to indicate direct quotes from your manuscript), or elaborate on this item by providing additional information not in the ms, or briefly explain why the item is not applicable/relevant for your study

"Rather, improvement was seen at 8 weeks in the Link group that provided only psychological healthcare information. However, it is difficult to determine whether the mental diary has an adverse effect on mood management because the number of records was low between 4 and 8 weeks. Other apps specializing in symptom management [38, 39] including psychological coping strategies for depression and anxiety severity, help reduce psychological distress. User-customized psychiatric content beyond a mental diary is suggested for managing depressed moods.", "Challenges of app improvement
Only one in ten participants maintained their daily visits after completion of the intervention, between 4 and 8 weeks.~~~Therefore, it is natural that PLWHs desire to stay up-to-date on the treatment, and the ESSC app needs to construct additional news sessions on the latest information." "Implications for mHealth adoption
The Link group, who could access digital information from individual text messages without signing up for the app, showed significantly improved self-efficacy in managing depression/mood, fatigue, and getting support/help. ~~"

20) Trial limitations, addressing sources of potential bias, imprecision, and, if relevant, multiplicity of analyses

20-i) Typical limitations in ehealth trials

Typical limitations in ehealth trials: Participants in ehealth trials are rarely blinded. Ehealth trials often look at a multiplicity of outcomes, increasing risk for a Type I error. Discuss biases due to non-use of the intervention/usability issues, biases through informed consent procedures, unexpected events.

	1	2	3	4	5	
subitem not at all important	○	○	○	○	◉	essential

선택해제

Does your paper address subitem 20-i? *

Copy and paste relevant sections from the manuscript (include quotes in quotation marks "like this" to indicate direct quotes from your manuscript), or elaborate on this item by providing additional information not in the ms, or briefly explain why the item is not applicable/relevant for your study

"Limitations
There are several limitations in interpreting the results. First, the inclusion criteria for smartphone users may not be representative of the generalized findings for PLWHs. To ensure representativeness and health equity, further studies are needed to enhance community-based enrollment without limiting smartphone literacy. Second, the intervention period of 4 weeks is short to fully investigate the effectiveness and usability of the app. As the intervention was a structured, information-driven online delivery to improve self-efficacy for HIV management, it was not appropriate to set a long delivery period for informational mHealth. To determine the usefulness of the ESSC app, it is necessary to evaluate its long-term effectiveness after app improvements."

21) Generalisability (external validity, applicability) of the trial findings

NPT: External validity of the trial findings according to the intervention, comparators, patients, and care providers or centers involved in the trial

21-i) Generalizability to other populations

Generalizability to other populations: In particular, discuss generalizability to a general Internet population, outside of a RCT setting, and general patient population, including applicability of the study results for other organizations

	1	2	3	4	5	
subitem not at all important	○	○	○	○	◉	essential

선택해제

Does your paper address subitem 21-i?

Copy and paste relevant sections from the manuscript (include quotes in quotation marks "like this" to indicate direct quotes from your manuscript), or elaborate on this item by providing additional information not in the ms, or briefly explain why the item is not applicable/relevant for your study

"Limitations
There are several limitations in interpreting the results. First, the inclusion criteria for smartphone users may not be representative of the generalized findings for PLWHs. To ensure representativeness and health equity, further studies are needed to enhance community-based enrollment without limiting smartphone literacy."

21-ii) Discuss if there were elements in the RCT that would be different in a routine application setting

Discuss if there were elements in the RCT that would be different in a routine application setting (e.g., prompts/reminders, more human involvement, training sessions or other co-interventions) and what impact the omission of these elements could have on use, adoption, or outcomes if the intervention is applied outside of a RCT setting.

	1	2	3	4	5	
subitem not at all important	○	○	○	◉	○	essential

선택해제

Does your paper address subitem 21-ii?

Copy and paste relevant sections from the manuscript (include quotes in quotation marks "like this" to indicate direct quotes from your manuscript), or elaborate on this item by providing additional information not in the ms, or briefly explain why the item is not applicable/relevant for your study

"Second, the intervention period of 4 weeks is short to fully investigate the effectiveness and usability of the app. As the intervention was a structured, information-driven online delivery to improve self-efficacy for HIV management, it was not appropriate to set a long delivery period for informational mHealth. To determine the usefulness of the ESSC app, it is necessary to evaluate its long-term effectiveness after app improvements."

OTHER INFORMATION

23) Registration number and name of trial registry

Does your paper address CONSORT subitem 23? *

Copy and paste relevant sections from the manuscript (include quotes in quotation marks "like this" to indicate direct quotes from your manuscript), or elaborate on this item by providing additional information not in the ms, or briefly explain why the item is not applicable/relevant for your study

"This study was approved by the Institutional Review Board of the first author's affiliation (4-2022-1192) and the protocol was registered with the Clinical Research Information Service (KCT0008014)"

24) Where the full trial protocol can be accessed, if available

Does your paper address CONSORT subitem 24? *

Cite a Multimedia Appendix, other reference, or copy and paste relevant sections from the manuscript (include quotes in quotation marks "like this" to indicate direct quotes from your manuscript), or elaborate on this item by providing additional information not in the ms, or briefly explain why the item is not applicable/relevant for your study

"This study was approved by the Institutional Review Board of the first author's affiliation (4-2022-1192) and the protocol was registered with the Clinical Research Information Service (KCT0008014)"

25) Sources of funding and other support (such as supply of drugs), role of funders

Does your paper address CONSORT subitem 25? *

Copy and paste relevant sections from the manuscript (include quotes in quotation marks "like this" to indicate direct quotes from your manuscript), or elaborate on this item by providing additional information not in the ms, or briefly explain why the item is not applicable/relevant for your study

"Funding-
This work was supported by the National Research Foundation of Korea grant funded by the South Korean Government (Ministry of Science and ICT) (no. 2020R1A2C101081713) (principal investigator [PI]: Gwang Suk Kim). The corresponding author received scholarships from the Brain Korea 21 FOUR Project funded by the National Research Foundation of Korea, Yonsei University College of Nursing. "

X27) Conflicts of Interest (not a CONSORT item)

X27-i) State the relation of the study team towards the system being evaluated

In addition to the usual declaration of interests (financial or otherwise), also state the relation of the study team towards the system being evaluated, i.e., state if the authors/evaluators are distinct from or identical with the developers/sponsors of the intervention.

	1	2	3	4	5	
subitem not at all important	○	○	○	○	◉	essential

선택해제

Does your paper address subitem X27-i?

Copy and paste relevant sections from the manuscript (include quotes in quotation marks "like this" to indicate direct quotes from your manuscript), or elaborate on this item by providing additional information not in the ms, or briefly explain why the item is not applicable/relevant for your study

"Conflicts of Interest-none declared"

About the CONSORT EHEALTH checklist

As a result of using this checklist, did you make changes in your manuscript? *

○ yes, major changes

◉ yes, minor changes

○ no

What were the most important changes you made as a result of using this checklist?

Some information was added to the abstract

How much time did you spend on going through the checklist INCLUDING making changes in your manuscript *

spend 3 hours for completing the checklist

As a result of using this checklist, do you think your manuscript has improved? *

- (●) yes
- ( ) no
- ( ) 기타:

Would you like to become involved in the CONSORT EHEALTH group?

This would involve for example becoming involved in participating in a workshop and writing an "Explanation and Elaboration" document

- ( ) yes
- (●) no
- ( ) 기타:

선택해제

Any other comments or questions on CONSORT EHEALTH

내 답변

STOP - Save this form as PDF before you click submit

To generate a record that you filled in this form, we recommend to generate a PDF of this page (on a Mac, simply select "print" and then select "print as PDF") before you submit it.

When you submit your (revised) paper to JMIR, please upload the PDF as supplementary file.

Don't worry if some text in the textboxes is cut off, as we still have the complete information in our database. Thank you!

Final step: Click submit !

Click submit so we have your answers in our database!

제출

양식 지우기

Google Forms를 통해 비밀번호를 제출하지 마세요.

이 콘텐츠는 Google이 만들거나 승인하지 않았습니다. 악용사례 신고 - 서비스 약관 - 개인정보처리방침

Google 설문지
